# Skeletal Involvement in Systemic Mastocytosis: Pathophysiology, Clinical Management, Standards of Care, and Novel Therapeutic Strategies

**DOI:** 10.3390/cells15030307

**Published:** 2026-02-06

**Authors:** Manlio Fazio, Adele Bottaro, Maria Elisa Nasso, Fabio Stagno, Alessandro Allegra

**Affiliations:** Division of Hematology, Department of Human Pathology in Adulthood and Childhood “Gaetano Barresi”, University of Messina, Via Consolare Valeria 1, 98125 Messina, Italy; manliofazio@hotmail.it (M.F.); adelebottarp15@gmail.com (A.B.); mariaelisanasso@gmail.com (M.E.N.); stagnof@unime.it (F.S.)

**Keywords:** systemic mastocytosis, RANK-RANKL-OPG, Wnt-signaling, KIT inhibitors, avapritinib, romosozumab, precision medicine, bone remodeling, mast cell biology

## Abstract

**Highlights:**

**What are the main findings?**

- Neoplastic mast cells disrupt bone homeostasis by upregulating RANKL and secreting Wnt antagonists (DKK1 and sclerostin), cytokines, and microRNAs that inhibit osteoblast differentiation and promote osteoclastogenesis.
- KIT-targeting inhibition with selective tyrosine kinase inhibitors (TKIs), such as avapritinib, harbors the potential to reverse these molecular alterations and restore balanced bone remodeling by reducing mast cell burden in systemic mastocytosis.

**What are the implications of the main findings?**

- Targeting KIT-driven signaling offers a mechanism-based strategy to treat SM-related bone disease, addressing both osteoporosis and osteosclerosis.
- Understanding mast cell-mediated modulation of RANKL/OPG and Wnt pathways opens avenues for precision therapies, including KIT inhibitors and Wnt modulators.

**Abstract:**

Systemic mastocytosis comprises a group of clonal mast cell disorders characterized by multisystem involvement. Bone involvement represents a major source of morbidity, particularly in young men affected by indolent systemic mastocytosis. This review provides an integrated and up-to-date overview of SM-related bone disease. We dissect the dual and context-dependent role of mast cells in bone remodeling, detailing how they promote osteoclastogenesis, suppress osteoblast function, and, in advanced disease, drive osteosclerosis. We critically appraise available treatments, including classic anti-resorptive therapy and emerging anabolic strategies. We further discuss the transformative impact of KIT-directed tyrosine kinase inhibitors, particularly avapritinib, which has demonstrated for the first time the ability to reverse not only osteoporosis but also osteosclerosis. Finally, we explore the emerging role of machine learning models in SM, proposing their application to individualized prediction of osteoporosis and fracture risk in SM. By bridging clinical care, bone biology, and therapeutic advances, this review underscores the need for a paradigm shift in which SM-related bone disease is recognized as a dynamic process requiring early identification, integrated risk stratification, and coordinated use of anti-resorptive, disease-modifying, and data-driven precision approaches to prevent fractures and improve long-term outcomes and quality of life in this delicate category of patients.

## 1. Introduction

### General Considerations on Mastocytosis

Systemic mastocytosis (SM) encompasses a heterogeneous spectrum of rare clonal disorders characterized by the proliferation and accumulation of abnormal mast cells (MCs) in one or multiple organs. According to the 5th edition of the World Health Organization (WHO), SM is divided into further subcategories based on disease burden, organ damage, and associated hematologic neoplasms [1,2,3,4]. The pathogenic hallmark of SM lies in the constitutive activation of the KIT tyrosine kinase, most commonly due to the D816V mutation, which drives MCs’ survival, mediator release, and tissue infiltration. Other potential driver mutations have also been studied [5,6].

Among target organs, the skeleton represents one of the most frequently and profoundly affected sites. Bone manifestations range from severe osteoporosis with fragility fractures to diffuse osteosclerosis or even mixed lesions within the same patient, reflecting the complex interplay between mast cells (MCs) and the bone microenvironment, including osteoclasts, osteoblasts, mesenchymal stem cells (MSCs), and the cytokine milieu. This pathophysiological heterogeneity translates into significant diagnostic and therapeutic challenges. Notably, up to one-third of SM patients presenting with fragility fractures display T-scores above −2.5 SD, indicating that deterioration of bone quality, rather than bone density alone, plays a central role.

In the past, given the rarity of SM, evidence guiding the management of SM-related bone disease largely relied on small retrospective analyses. Nevertheless, in recent years, therapeutic innovation has reshaped the landscape. The advent of targeted tyrosine kinase inhibitors (TKIs) has offered not only symptomatic relief but also potential disease-modifying effects, including improvements in bone mineral density (BMD), osteosclerosis, and bone marrow MC infiltration. This review provides an updated and integrative overview of bone involvement in SM, spanning epidemiology and pathophysiology to current and emerging therapeutic approaches. We also emphasize the potential contribution of machine learning and imaging-based models to risk stratification, prediction of skeletal complications, and therapeutic personalization.

To develop this narrative review, a comprehensive literature search was conducted using the PubMed/MEDLINE database to identify relevant studies addressing bone involvement in SM. The search covered a 30-year time frame, from January 1995 to December 2025. The search strategy combined Medical Subject Headings (MeSH) terms and free-text keywords related to SM and skeletal disease. Core search terms included the following: “systemic mastocytosis,” “osteoporosis,” “fragility fractures,” “osteosclerosis,” and “bone turnover.” Additional searches incorporated therapeutic keywords including “bisphosphonates,” “interferon-alpha,” “denosumab,” “romosozumab,” and “tyrosine kinase inhibitors.” To capture advances in disease stratification and management, further searches included terms related to “*KITD816V* allele burden,” “prognostic scores,” “fracture risk prediction,” “imaging-based bone assessment,” and “machine learning.” Reference lists of key articles and clinical guidelines were manually reviewed to identify additional relevant publications. Eligible sources included original clinical studies, cohort analyses, clinical trials, translational studies, consensus recommendations, and reviews published in peer-reviewed journals and written in English. Case reports were considered when addressing rare skeletal phenotypes or providing mechanistic insights.

## 2. Physio-Pathological Role of Mast Cells in Bone Metabolism

### 2.1. Osteo-Resorptive Function

Mast cells are increasingly recognized as central regulators of skeletal homeostasis, influencing both physiological bone remodeling and pathological bone turnover. Their impact is bidirectional. More specifically, they can either drive profound bone loss and microarchitectural deterioration or act as essential contributors to normal fracture healing, angiogenesis, and bone regeneration. This duality reflects their complex interactions with the BM microenvironment and all its components: osteoclasts, osteoblasts, immune cells, endothelial cells, and stromal cells [7].

Before focusing on SM, it is worth recalling that bone remodeling is governed by a finely tuned balance between osteoblast-mediated bone formation and osteoclast-driven resorption. These processes are primarily regulated by two interconnected pathways. The first is a system composed of three members: the Receptor Activator of the Nuclear Factor κB (RANK), its ligand (RANKL), and osteoprotegerin (OPG); the second is composed of Wingless (Wnt) signaling molecules and β-catenin, which acts as a transcription co-activator [8].

The RANK-RANKL represents the central driver of osteoclast differentiation and activation, whereas OPG, acting as a soluble decoy receptor, counterbalances this process by limiting RANKL availability and restraining bone resorption. Dysregulation of this axis (that is, if the RANKL/OPG ratio is high), rather than its physiological function per se, underlies pathological bone loss in several disease settings, including SM [9,10]. Parallel to this, the Wnt/β-catenin pathway cooperates with bone morphogenetic proteins (for example, BMP 2 and BMP7) to promote osteogenesis [11]. When Wnt proteins bind to the LRP5/6+Frizzled receptor on the membrane of osteoblasts and stromal cells, β-catenin is not degraded, activates osteogenic genes (for example, *RUNX2*, OPG), and determines the following: (1) increased osteoblastic differentiation (which translates into bone formation); (2) reduced osteoclastic activity (due to higher OPG levels) [11]. On the other side, Dickkopf-1 (DKK1) and sclerostin (SOST, produced by osteocytes) represent the antagonists of the Wnt pathway. They bind to LRP5/6 and thus prevent Wnt from activating the receptor and attenuate β-catenin signaling. Furthermore, soluble Frizzled-related proteins (sFRPs) are extracellular Wnt inhibitors that act as decoy receptors that contain soluble Wnt-binding frizzled domains. This mechanism further attenuates Wnt-LRP5/6 signaling and prevents β-catenin stabilization and nuclear translocation in osteoblast precursors. This complementary layer of Wnt antagonism reinforces osteoblast suppression and amplifies the negative remodeling balance [12].

This leads to a negative turnover balance, with reduced osteoblast activation and numbers [13]. Furthermore, DKK1 and SOST can also interfere with BMP signaling [13]. Fluctuations in these inhibitors are known to produce divergent bone phenotypes, ranging from severe osteoporosis to high-bone-mass disorders [14].

Both RANKL/OPG and Wnt/β-catenin pathways can be modulated by MCs in pathological and physiological conditions. In detail, pathologic MCs are able to release RANKL, leading to elevated circulating concentrations. Interestingly, the same phenomenon occurs with OPG [15]. However, despite increased OPG, which might be expected to counterbalance osteoclastogenesis, its effect turns out to be insufficient due to an overwhelming excess of RANKL and other pro-resorptive cytokines. On the other side, MCs can directly produce DKK1 and SOST and subsequently provide another mechanism for bone demineralization [16,17]. Consistently, MC-mediated inhibition of Wnt/β-catenin and BMP signaling in osteoblast-lineage cells not only suppresses osteoblast differentiation through *RUNX2* downregulation but also secondarily reduces osteoblast-derived OPG production, thereby further weakening the physiological restraint on osteoclast activation and amplifying net bone resorption [18].

In physiological conditions, MC-derived histamine, IL-6, and TNF-α orchestrate osteoclastogenesis by enhancing RANKL expression on osteoblasts and stromal cells and modestly suppressing OPG production. Furthermore, MC-derived proteases, particularly tryptase, activate latent TGF-β and matrix metalloproteinases (MMPs), participating in extracellular matrix remodeling and coupling resorption to formation [19]. Meanwhile, MC-derived heparin and vascular endothelial growth factors (VEGF) support microvascular homeostasis, which is essential for nutrient transport to bone-forming sites [20]. These mechanisms appear consistently dysregulated in the SM setting, where gain-of-function KIT mutations (typically *KITD816V*) result in constitutive MC activation, survival, and accumulation within the BM. Thus, neoplastic MCs secrete large quantities of histamine, tryptase, IL-1β, IL-6, TNF-α, prostaglandins, and leukotrienes, all of which converge to amplify osteoclastogenesis and suppress osteoblast function [21,22].

Adding further complexity, new evidence indicates that MCs secrete microRNAs such as *miR-23a* and *miR-30a*, which inhibit osteoblast differentiation and mineralization, representing a highly efficient intercellular communication mechanism by which MCs can exert sustained, post-transcriptional repression of osteogenic programs in neighboring stromal and osteoblast progenitor cells [23,24].

**Figure 1:**
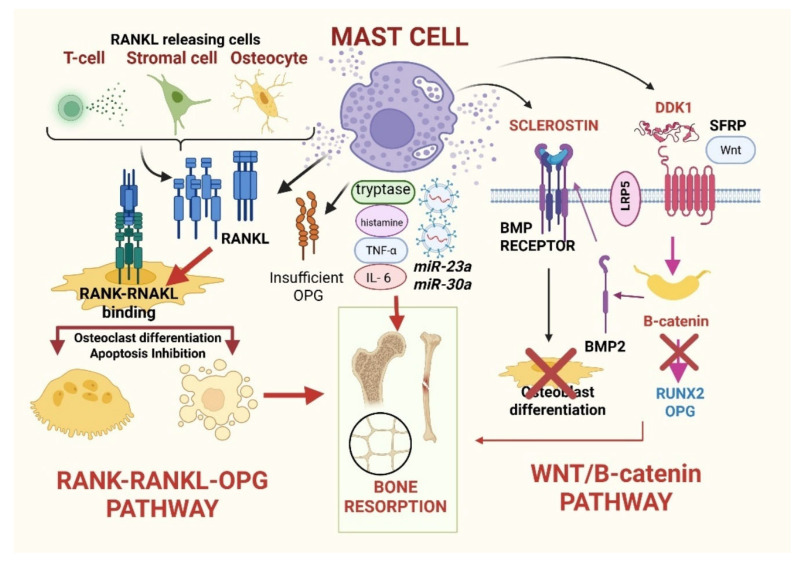
Osteo-resorptive function of mast cells. Activated MCs promote bone resorption through converging mechanisms that disrupt the balance between osteoclast and osteoblast activity. On the osteoclastogenic side, MCs directly and indirectly enhance RANK-RANKL signaling by contributing to RANKL availability from immune cells, stromal cells, and osteocytes, while OPG activity is insufficient to counterbalance RANK-RANKL binding on osteoclast precursors. In parallel, activated MCs release pro-osteoclastogenic mediators and secrete extracellular vesicles containing microRNAs, further amplifying osteoclast formation, survival, and bone resorption. Concomitantly, MCs impair osteoblast differentiation and function by inhibiting the canonical Wnt/β-catenin pathway through increased expression of sclerostin and DKK1, interference with Wnt ligand binding to the LRP5/6 co-receptor complex (including soluble Wnt antagonists such as sFRP), and downstream reduction in β-catenin signaling. Additional suppression of osteoblastogenesis occurs via altered BMP receptor signaling, reduced BMP2 activity, downregulation of the osteogenic transcription factor *RUNX2*, and decreased OPG production. Abbreviations: RANK (Receptor Activator of Nuclear Factor κB); RANKL (Receptor Activator of Nuclear Factor κB Ligand); OPG (Osteoprotegerin); miR (microRNA); SFRP (Secreted Frizzled-Related Protein); LRP5 (Low-Density Lipoprotein Receptor–Related Protein 5); DKK1 (Dickkopf-related protein, most commonly Dickkopf-1); BMP (Bone Morphogenetic Protein).

Figure 1 summarizes the mechanistic aspects described in this section and shows how MCs modulate the main molecular patterns related to bone homeostasis.

### 2.2. Osteo-Genetic Function

As mentioned in the previous section, MCs are also involved in ossification. In physiological conditions, this action leads to bone repair and fracture healing; in pathological conditions, it causes excessive bone matrix deposition and causes osteosclerosis. In addition, in the SM setting, it is not uncommon to find a coexistence of both phenotypes (osteoporotic and osteosclerotic) [25]. Mechanistically, several convergent pathways likely drive focal osteoblastic overactivity and matrix deposition. Activated MCs secrete proteases (chymase and tryptase) that can convert latent TGF-β into its active form; active TGF-β promotes fibroblast-to-osteoblast trans-differentiation and extracellular matrix production through SMAD2/3, and it synergizes with BMP signaling to upregulate *RUNX2* and Sp7 (a transcription factor essential for bone formation) in osteoprogenitors [26,27]. MC-derived fibroblast growth factor-2 (FGF-2) engages FGFR/ERK and PI3K pathways in stromal cells to expand osteoprogenitor pools and increase collagen synthesis, while IL-11, acting through gp130/STAT3, promotes osteoblast differentiation and survival and can tip remodeling toward bone formation when chronically elevated [20].

Key microenvironmental modifiers, particularly marrow perfusion and oxygen availability, are likely to shape the divergent bone phenotypes observed in SM. Reduced perfusion can create localized hypoxic niches, stabilizing HIF-1α in stromal cells and enhancing VEGF-driven angiogenesis, which in turn facilitates osteoblast recruitment and mineral deposition [16]. Concurrently, SM-associated fibrosis decreases marrow hydraulic conductivity, effectively sequestering osteogenic growth factors within restricted microdomains. The resulting focal enrichment of MC-derived TGF-β, FGF-2, and IL-11, together with impaired perfusion dynamics, may generate discrete pockets of excessive osteoblastic activity, manifesting as sclerotic lesions, immediately adjacent to regions dominated by osteoclastic resorption and osteoporosis. This spatial heterogeneity provides a mechanistic framework for the characteristic coexistence of osteosclerosis and bone loss within the same skeleton in SM [25].

**Figure 2:**
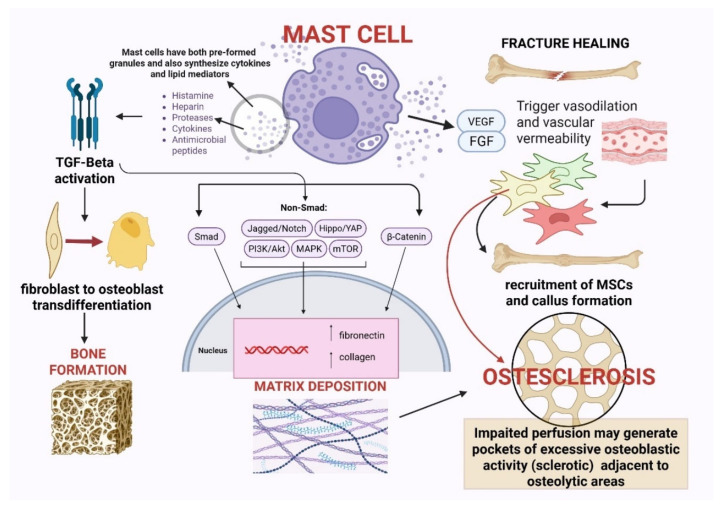
Osteogenic function of mast cells. This image shows the major pathways through which MCs contribute to bone formation and fracture healing in physiological conditions and to osteosclerosis in the SM setting. Under physiological conditions, MCs contribute to bone formation and fracture repair through tightly regulated paracrine and matrix-modulating mechanisms. Activated MCs release preformed granule mediators as well as newly synthesized cytokines, growth factors, and lipid mediators, which collectively modulate the bone microenvironment. MC-derived TGF-β promotes fibroblast-to-osteoblast trans-differentiation and osteoprogenitor activation, supporting bone formation. In parallel, MCs activate both canonical and non-canonical signaling pathways in MSCs and osteoblast precursors, enhancing extracellular matrix deposition through increased collagen and fibronectin synthesis. During fracture healing, MCs secrete angiogenic factors such as VEGF and FGF, promoting vasodilation, increased vascular permeability, MSC recruitment, callus formation, and coordinated bone repair. Crosstalk with β-catenin signaling further supports osteoblast differentiation and matrix maturation. In the setting of SM, persistent MC activation and accumulation disrupt the spatial and temporal regulation of these osteogenic pathways. This phenomenon may generate focal or diffuse osteosclerosis, often juxtaposed to osteolytic regions. Abbreviations: TGF-β (transforming growth factor Beta); VEGF/FGF (vascular endothelial/fibroblast growth factor); MSC (mesenchymal stem cell).

As shown in in vivo models, fracture healing illuminates the dual and context-dependent roles of MCs more clearly [22]. The normal repair process comprises inflammatory, reparative, and remodeling phases, and MCs contribute to each via rapid sensing and mediator release. MCs detect tissue damage through complement receptors and damage-associated molecular patterns and (DAMP)-sensing pathways and respond to hypoxia and mechanical disruption by degranulating and releasing histamine, tryptase, TNF-α, IL-6, and IL-1β. These mediators promote neutrophil and monocyte recruitment, polarize macrophages toward repair-supporting phenotypes (via IL-1/IL-6/TNF signaling), and set up the matrix-remodeling program required for callus formation [17,28,29,30]. Furthermore, MC-derived VEGF, FGF-2, and histamine promote endothelial permeability and neovascularization, processes that are indispensable for the recruitment of MSCs and the formation of the cartilaginous soft callus [28,29]. Understanding the molecular determinants of these divergent bone phenotypes offers opportunities for targeted therapies, including KIT inhibitors, anti-RANKL therapy, Wnt-pathway modulators, and MC-stabilizing strategies, which may redefine future management of MC-driven bone disease. It should be specified that a critical methodological limitation in the current understanding of MCs’ bone interactions is that a great part of mechanistic data derives from murine models and mouse-derived MCs, which may not fully recapitulate the behavior of human MCs in physiological or pathological settings. Although mouse models have been useful for delineating fundamental pathways regulating osteoclastogenesis, osteoblast differentiation, and fracture repair, important interspecies differences exist in MC ontogeny, receptor expression, mediator content, and functional responses. Moreover, the BM microenvironment in humans, particularly in clonal MC disorders such as SM, is shaped by complex interactions between neoplastic MCs, stromal cells, osteoblasts, osteoclasts, and endothelial cells that cannot be fully modeled in mice. Therefore, while murine studies provide an essential biological framework, findings should be interpreted with caution, and there is a pressing need for translational studies leveraging primary human MCs, human BM-derived stromal systems, and clinical correlates to validate the relevance of these pathways in human skeletal disease. The various mechanistic insights described in this section are summarized in Figure 2.

### 2.3. Calcium and PI3K/Akt/mTOR Signaling as Integrative Pathogenic Axes in Systemic Mastocytosis

Calcium ion (Ca^2+^) signaling represents a central and evolutionarily conserved regulatory mechanism governing MC activation, mediator release, and survival, and its dysregulation plays a direct pathogenic role in SM [31]. In MCs, antigen-dependent and antigen-independent stimuli converge on Ca^2+^ influx through plasma membrane channels and Ca^2+^ release from intracellular stores, particularly the endoplasmic reticulum (ER), triggering degranulation, cytokine synthesis, lipid mediator production, and activation of downstream pathways such as protein kinase C (PKC), MAPK, and NF-κB. As highlighted by Lobefalo et al., Ca^2+^ fluxes act as universal signal integrators in immune cells, coupling environmental stimuli to inflammatory responses, cytokine release, and stress-adaptive programs through ER-mitochondrial crosstalk, PKC activation, and modulation of innate immune receptors. In SM, constitutive *KIT* activation amplifies Ca^2+^-dependent signaling, lowering activation thresholds and promoting chronic MC hyper-responsiveness, spontaneous mediator release, and sustained inflammatory signaling. This aberrant Ca^2+^ homeostasis likely contributes not only to systemic symptoms and anaphylaxis risk but also to MC-driven bone remodeling abnormalities, as Ca^2+^-dependent pathways regulate osteoclastogenic cytokine secretion, RANKL expression, and osteoblast–osteoclast coupling. Moreover, emerging evidence that pharmacological modulation of Ca^2+^ signaling can attenuate excessive immune activation raises the possibility that Ca^2+^-targeted strategies may represent an unexplored adjunctive approach in SM, warranting further investigation in human disease models [31]. Beyond Ca^2+^ signaling, constitutive activation of the PI3K/Akt/mTOR axis represents a central driver of MCs’ survival, proliferation, and functional activation in SM, largely downstream of oncogenic *KIT* mutations [32]. Mutated *KIT* engages multiple intracellular cascades, among which PI3Kβ-dependent signaling plays a pivotal role in sustaining MCs’ viability, metabolic fitness, and resistance to apoptosis, while mTOR integrates growth factor and nutrient signals to promote protein synthesis, cell growth, and inhibition of autophagy. As highlighted by Avivar-Valderas, PI3Kβ and mTOR are also key regulators of immune homeostasis, linking inflammatory signaling, metabolic adaptation, and host defense mechanisms. In SM, chronic activation of this pathway likely contributes to persistent MC accumulation, exaggerated mediator release, and immune dysregulation, while simultaneously influencing the bone microenvironment through enhanced cytokine production, RANKL expression, and altered osteoblast–osteoclast coupling [32]. The intersection between PI3K/Akt/mTOR signaling, immune activation, and tissue remodeling provides a mechanistic framework to understand disease chronicity and therapeutic resistance and supports growing interest in pathway-targeted approaches, including *KIT* inhibitors and mTOR-modulating strategies, as potential interventions in selected SM phenotypes.

## 3. Epidemiology of Bone Disease in Systemic Mastocytosis

Mastocytosis has an annual incidence of 0.77–2.77 per 100.000 and a prevalence of 9.6–17.2 per 100.000, although this value is likely underestimated due to nonspecific symptoms and low diagnostic awareness [33]. Bone involvement occurs in approximately 70% of SM patients, most commonly affecting the spine, with osteopenia, osteoporosis, and fractures reported in up to 40% of cases [16]. The burden of skeletal disease varies markedly across the SM spectrum, with ISM and BMM showing the highest frequency of osteoporosis and fragility fractures, while AdvSM more often exhibits osteosclerosis or even increased BMD [34]. Among all SM variants, ISM has historically been the subtype most closely associated with bone loss [16,35]. Several cohort studies consistently report osteoporosis in 20–38% of ISM patients [36,37,38,39].

Importantly, if the paradigm of primary osteoporosis is the postmenopausal woman or the senile individual, SM represents a disproportionately common cause of secondary osteoporosis in young men (an otherwise low-risk demographic) [37]. Evidence from a German center showed that among 1.374 patients evaluated for osteoporosis, systematic BM biopsy identified indolent SM in nearly 0.5% of all cases and in >5% of young men with otherwise unexplained osteoporosis [37]. BMM, now recognized as a distinct entity separate from ISM, presents with a similar skeletal phenotype despite lacking skin lesions and B-findings [40]. In expert centers, BMM accounts for about 17% of non-advanced SM and shows osteoporosis and vertebral fractures at rates comparable to ISM [41]. Fragility, and especially vertebral fractures, represent the most clinically disabling consequence of SM-related bone disease [42]. Lifetime vertebral fracture rates approach 30–40% in ISM cohorts, and as previously specified, a significant proportion of fractures occur in patients with T-scores ≥ 2.5, revealing a certain disconnect between BMD and skeletal fragility [42]. This discrepancy reflects inherent microarchitectural instability and MC-mediated bone remodeling abnormalities not captured by dual-energy X-ray absorptiometry (DXA).

Contrasting with ISM and BMM, AdvSM (all three subtypes: ASM, SM-AHN, and MCL) shows a dramatically different skeletal phenotype. Registry data demonstrate osteoporosis in only ~6% of AdvSM patients [38]. Instead, the majority display normal or increased BMD, and osteosclerotic lesions are frequent, reflecting high MC burden and altered cytokine milieu [38]. In some cohorts, increased BMD or osteosclerosis was observed in ~75% of AdvSM patients [38]. These patients may suffer from bone pain due to increased remodeling, focal fibrosis, and high marrow turnover despite elevated BMD values. Recent studies highlight that Monoclonal Mast Cells Activation Syndrome (MMAS), while less severe than SM, is also associated with increased rates of osteopenia and osteoporosis, albeit typically with additional conventional risk factors [43]. MMAS is a clonal MC disorder characterized by episodic MC activation and evidence of MC clonality (for example, CD25 positivity) but without fulfilling the histological or clinical criteria for SM.

Unlike SM, MMAS exhibits low MC burden, normal or minimally elevated tryptase, and absence of BM aggregates or organ involvement. *KITD816V* is often present but at a very low allele burden [44]. Bone involvement can occur but is generally milder than in ISM, with definitively lower rates of vertebral fracture cascades [42]. Lastly, bone pain is a clinical manifestation reported in up to 54% of SM patients. Patients commonly experienced poorly localized bone pain (femur, pelvic bones, joints, and smaller bones, skull, spine, ribs, and hands), which can be described as severe or intolerable in 18% of instances [35,45]. This potentially invalidating symptom is also encountered in those individuals who present mixed skeletal phenotypes (coexisting osteoporotic and osteosclerotic lesions within the same individual). Some of the most important studies that helped to define the epidemiological data reported in this section are summed up in Table 1.

## 4. Comprehensive Approach to Bone Health Assessment

### 4.1. Metabolic Work-Up

In patients with SM, whether ISM or AdvSM, a systematic and stepwise evaluation of bone health is essential. Clinical evaluation is crucial, with attention to sarcopenia, balance, vision, medications (that is, steroids), and prior falls, complemented by simple functional tests in older patients or those with prior fragility fractures. All patients should receive supportive measures (that is, adequate calcium and vitamin D intake, weight-bearing exercise, avoidance of smoking and alcohol, and fall prevention strategies). A comprehensive metabolic workup should include all those markers that are endorsed by international guidelines [International Osteoporosis Foundation (IOF)/International Federation of Clinical Chemistry and Laboratory Medicine (IFCC)] and are considered valuable adjuncts for monitoring bone changes over time or treatment response [46]:

- Serum calcium (Ca) and phosphorus (P) to exclude metabolic bone disease.
- 25-hydroxyvitamin D to detect deficiency that can exacerbate bone turnover.
- Bone turnover markers (BMTs) such as bone-specific alkaline phosphatase (BSAP) and serum C-terminal telopeptide of type I collagen (CTX) to estimate osteoblastic activity and osteoclast-driven bone resorption, respectively. Regarding BSAP, it is also included as a parameter in SM prognostic scores such as the International Prognostic Scoring System for SM (IPSM) and the Global Prognostic Score for mastocytosis (GPSM) [47,48].

Interestingly, BSAP and CTX levels are elevated in both osteoporotic and osteosclerotic conditions. In the former, CTX increases due to hyperactive osteoclasts, while BSAP rises because osteoblasts attempt to compensate; in the latter, BSAP is elevated as a marker of excessive bone formation, whereas CTX increases as a compensatory mechanism aimed at reducing the excessive bone deposition [49].

- Additional laboratory tests: parathyroid hormone (PTH), renal function, and albumin may be considered to rule out other causes of secondary osteoporosis.
- Baseline serum tryptase (bST) is useful to better characterize SM subtypes, and by extension, the bone disease phenotype. In other words, higher levels of bST are usually found in AdvSM which presents with more aggressive features and higher molecular burden (↑*KIT* VAF, additional mutations). In these cases, it is more common to find osteosclerotic bone alterations [50].
- DXA of the spine and hip remains the gold standard for BMD measurement (trabecular-rich sites which reflect BM tropism of clonal MCs and the metabolic activity of trabecular bone). In this regard, thoraco-lumbar spine radiographs should be performed at baseline and periodically, even in asymptomatic patients, as nearly 30% of vertebral fractures are clinically silent [34]. Z-scores < −2 [standard deviation (SD) below the age- and gender-matched mean reference value) are more accurate and clinically meaningful than T-scores ≤ −2.5 (SD below the mean of young healthy adults) [50].

The latter statement is strongly corroborated by a study by Rossini et al., who provided an informative analysis of skeletal pathology in ISM, demonstrating that traditional WHO T-score criteria may under-detect disease-related bone loss. In the study, osteoporosis (T-score ≤ −2.5) was found in 20% of patients; however, when applying the more appropriate threshold of Z-score < −2, SM-related low BMD was identified in another 20% (including 9% of women and 28% of men) [51]. Crucially, the study showed that vertebral fractures occurred even in patients with Z > −2, again confirming that bone fragility in SM often exceeds what DXA captures. Vertebral fractures were present in 21% of the cohort, the majority occurring despite non-osteoporotic BMD, reinforcing that SM-related osteoporosis is a unique, high-risk skeletal phenotype characterized by an intrinsic bone fragility [50]. Additionally, men showed a disproportionately high burden of low BMD and vertebral fractures, further supporting the evidence that SM is overrepresented among men with apparent idiopathic osteoporosis [50].

Altogether, these data justify the concept that DXA assessment should be followed by proper vertebral imaging in order to detect eventual silent fractures. Van der Veer et al. made a comprehensive attempt to quantify fracture risk in ISM. In a cohort of 221 individuals with ISM, lifetime fracture documentation and detailed bone, metabolic, and clinical assessments demonstrated that 41% had experienced fragility fractures, while prospective follow-up (median 5.4 years) revealed 5- and 10-year risks of 23% and 31%, respectively [52]. Despite the high burden of fractures, only about 1/4 of patients who fractured had osteoporosis at diagnosis, highlighting once again the inadequacy of BMD alone as a predictor of skeletal fragility in ISM. Using multivariate Cox regression, the authors identified five independent predictors of future fragility fractures: male sex, elevated sCTX (Z-score ≥ +1), reduced hip BMD (T-score ≤ −1), absence of urticaria pigmentosa (UP), and alcohol consumption at diagnosis.

These variables (easily obtainable in clinical practice) were integrated into the MastFx score, a practical risk-stratification tool with good discriminatory accuracy (AUC = 0.80), which outperformed QFracture (a standard population-based risk calculator that significantly underestimated fracture risk in this younger, disease-specific population). This score enables early identification of high-risk individuals who may benefit from prompt therapeutic intervention to prevent fractures, underscoring the need for systematic skeletal evaluation and more intensive bone-protective strategies in ISM [52]. Although the MastFx score represents a promising risk stratification tool, it requires external validation in independent cohorts before its routine use in clinical practice can be recommended. Another delicate aspect is the longitudinal monitoring of those patients who are receiving anti-resorptive therapy (for example, bisphosphonates or denosumab).

**Figure 3:**
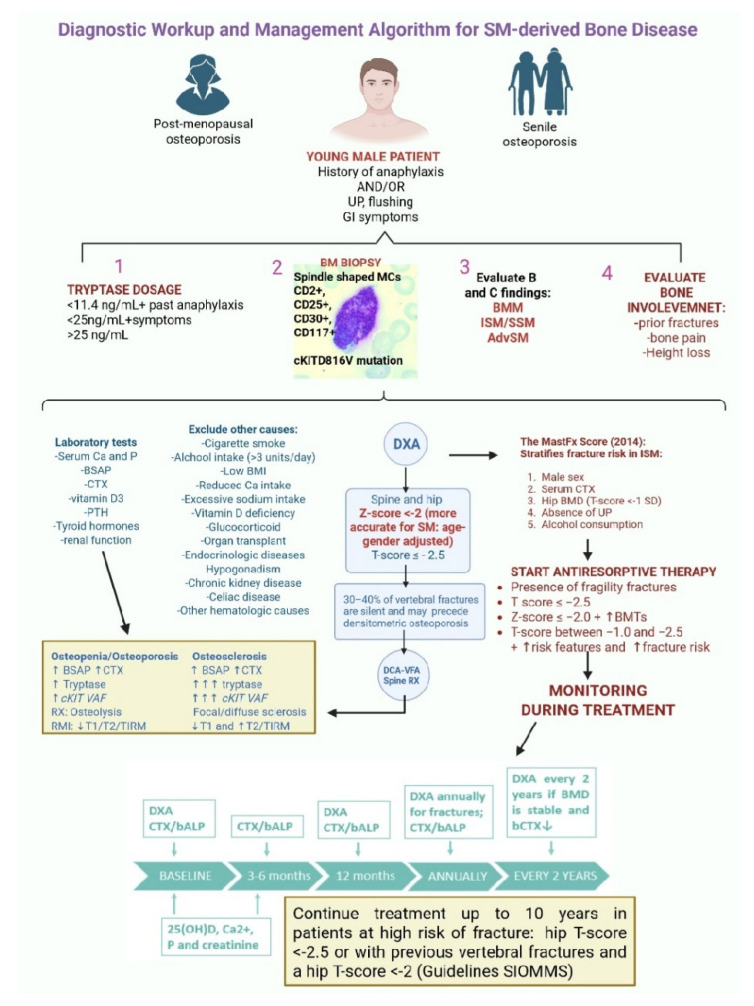
Diagnostic work-up and management algorithm for SM-derived bone disease. ABBREVIATIONS: SM (systemic mastocytosis), Ca (calcium), P (phosphorus), BSAP/bALP (bone-specific alkaline phosphatase), CTX (C-terminal telopeptide), PTH (parathormone), DXA (dual-energy X-ray absorptiometry), BMI (body mass index), VFA (vertebral fracture assessment), VAF (variant allele frequency), BMD (bone mineral density), UP (urticaria pigmentosa), BMT (bone turnover marker), RX (radiography), 25(OH)D (25 hydroxy vitamin D3—cholecalciferol), SIOMMS (Società Italiana dell’ Osteoporosi del Metabolismo Minerale e delle Malattie dello Scheletro).

The algorithm should include periodic DXA scans after 1 year and every 2 years in stable patients [more frequently (every year) in those with prior fractures and a higher risk of new lesions], together with repeated measurement of BTMs (every 3–6 months), which respond more rapidly than BMD to changes in bone metabolism and therapy. All these strategies combined can allow better management of this category of patients, remarkably improving their quality of life [53]. The whole diagnostic and management algorithm described in this section is schematized in Figure 3.

### 4.2. Radiological Assessment

Radiological manifestations in SM are highly heterogeneous:

- Solitary or multiple osteolytic lesions.
- Focal or multifocal osteosclerotic lesions often appear as numerous, rounded, sharply defined sclerotic foci in the axial skeleton, ribs, humerus, and femur.
- Diffuse osteosclerosis with marked trabecular thickening. Because this abnormality can closely mimic metastatic bone disease or osteopoikilosis [a rare, benign, genetic bone disorder causing dense, white spots (bone islands or enostoses) on X-rays, mainly near joints in long bones, hands, feet, and pelvis, looking like “spotted bone”], interpretation must always be integrated with the clinical picture, serum tryptase level, eosinophil count, and the absence of MRI features such as the “halo sign,” which is highly specific for metastases [54].

Conventional radiography is typically the first-line imaging modality, but its sensitivity is limited, especially for early disease or BM infiltration:

- PROS: X-rays may reveal multifocal or diffuse osteosclerosis, rarely lytic lesions, vertebral compression fractures, or cortical thinning in SM-related osteoporosis.
- CONS: Osteoporosis cannot be reliably detected radiographically until approximately 30% of bone mass is lost, and variations in exposure and soft-tissue thickness may obscure subtle trabecular abnormalities.

Despite these limitations, radiographs can raise suspicion for SM when interpreted in conjunction with systemic symptoms or elevated MC mediators [54].

#### Advanced Imaging to Assess Both Bone Disease and Bone Marrow Involvement

Beyond conventional radiography and DXA, advanced imaging modalities, including computed tomography (CT), magnetic resonance imaging (MRI), and positron emission tomography combined with CT (PET-CT), play complementary roles in the assessment of skeletal involvement in SM, allowing a more accurate characterization of bone phenotype, BM infiltration, and disease activity (Table 2).

Computed tomography, and especially low-dose whole-body CT, is particularly helpful for detecting focal abnormalities in patients without cutaneous involvement or when radiographic findings are equivocal. It substantially improves diagnostic sensitivity, providing high-resolution visualization of small lesions and detailed assessment of complex anatomical regions such as the thoracic spine and pelvis. Furthermore, it quantifies BM attenuation through Hounsfield units (HU), which correlates with MC burden and tends to be higher in AdvSM [55].

Magnetic resonance imaging remains the most sensitive modality for detecting BM infiltration, offering excellent tissue contrast and comprehensive evaluation of disease extent [55]. Typical MRI features include:

- Infiltrated marrow, characterized by T1 hypointensity and T2/TIRM hyperintensity.
- Osteosclerotic lesions showing diffuse T1 and T2/TIRM hypointensity.
- Osteolytic lesions that appear sharply demarcated with T1 hypointensity and T2/TIRM hyperintensity.

Whole-body MRI (WB-MRI) further enhances assessment by identifying reproducible marrow patterns associated with MC burden and prognosis. Treatment response may be suggested by increasing T1 and decreasing T2 signal intensity, although MRI is not yet validated for routine monitoring [55].

Radionuclide imaging (for example, bone scintigraphy) may demonstrate diffuse uptake patterns that evolve over time and reflect MCs’ expansion in the BM, but its specificity is limited. FDG-PET/CT has minimal utility in most SM subtypes. Indolent and smoldering AdvSM without associated hematologic neoplasms typically show absent or low-grade uptake, while increased FDG avidity is usually confined to SM-AHN or mast cell sarcoma (MCS). Consequently, PET/CT does not play a major role in the routine evaluation of SM-related bone disease [55].

## 5. Therapeutic Approaches to SM-Related Bone Disease

### 5.1. Vitamin D

Vitamin D represents a crucial immunomodulatory and osteoprotective factor at the intersection of MC biology, allergic inflammation, and bone metabolism. Beyond its classical role in calcium-phosphate homeostasis, vitamin D exerts direct inhibitory effects on MC activation through vitamin D receptor (VDR)-dependent mechanisms [56]. Experimental data demonstrate that vitamin D deficiency leads to spontaneous MC degranulation, whereas calcitriol stabilizes MCs by interfering with FcεRI-mediated signaling, reducing Syk phosphorylation, NF-κB and MAPK activation, and suppressing the transcription of pro-inflammatory mediators such as TNF-α. Vitamin D further promotes an anti-inflammatory immune milieu by enhancing IL-10 production, expanding regulatory T cells, inhibiting IgE synthesis, and inducing tolerogenic dendritic cells [56].

Importantly, MCs themselves can locally convert 25-hydroxyvitamin D into its active form via CYP27B1, establishing an autocrine loop that dampens IgE-mediated degranulation. In the context of bone disease, MC-derived mediators (histamine, tryptase, cytokines, RANKL) drive osteoclast activation and bone loss, a process exacerbated in vitamin D-deficient states. Collectively, these findings position vitamin D as a key regulator of MC stability and immune tolerance, with therapeutic potential to reduce mediator release, allergic manifestations, and SM-associated bone disease when appropriately supplemented, although both deficiency and excess appear detrimental, underscoring the need for individualized vitamin D optimization [56].

### 5.2. First-Line Approach: Bisphosphonates

Bisphosphonates (BPs) are potent anti-resorptive agents that exert their therapeutic effects primarily by targeting osteoclast-mediated bone resorption. Structurally analogous to inorganic pyrophosphate, BPs display a high affinity for hydroxyapatite crystals and selectively accumulate at sites of active bone remodeling. During bone resorption, osteoclasts internalize BPs, which then interfere with critical intracellular pathways. Nitrogen-containing BPs (for example, alendronate, pamidronate, and zoledronate), which represent the cornerstone of contemporary osteoporosis therapy, inhibit farnesyl pyrophosphate synthase within the mevalonate pathway. This blockade prevents the prenylation of small GTP-binding proteins (such as Ras, Rho, and Rac), which are essential for osteoclast cytoskeletal organization, vesicular trafficking, ruffled border formation, and survival, ultimately leading to profound suppression of osteoclast activity and apoptosis. The net effect is a progressive gain in BMD, particularly at trabecular-rich sites. Furthermore, BPs may exert indirect effects on the bone microenvironment by reducing the release of matrix-derived growth factors during resorption [57,58].

The safety and toxicity profile of BPs is generally favorable when used appropriately. Acute-phase reactions, characterized by transient flu-like symptoms, fever, myalgias, and arthralgias, commonly occur after the first IV administration and are attributed to cytokine release [59]. Unlike anaphylactic reactions, these manifestations are typically self-limited, do not involve hypotension, bronchospasm, or urticaria, and are attenuated by antipyretics. This distinction is particularly important in patients with SM, in whom heightened concern for anaphylaxis may lead to unnecessary discontinuation of an otherwise effective and safe anti-resorptive treatment. Gastrointestinal (GI) toxicity, including esophagitis and gastritis, is primarily associated with oral formulations. Renal toxicity, particularly acute tubular injury, represents a recognized risk of IV BPs, especially zoledronate, and mandates dose adjustment, adequate hydration, and avoidance in patients with severe renal impairment [60]. Hypocalcemia may occur, particularly in vitamin D-deficient states or in patients with high baseline bone turnover, underscoring the importance of calcium and vitamin D optimization prior to therapy [61].

Rare but serious long-term complications include osteonecrosis of the jaw (ONJ) and atypical femoral fractures (AFFs) [61]. In order to prevent ONJ, dental vigilance is mandatory before starting therapy. AFFs are thought to result from prolonged suppression of bone remodeling and microdamage accumulation and typically occur after long-term treatment exceeding five years. These risks have led to the concept of periodic treatment reassessment and, in selected low-risk patients, drug holidays.

#### 5.2.1. Zolendronate

The employment of BPs in SM-related osteoporosis has initially been described in the literature in case reports [62]. In 2014, Rossini et al. evaluated the effect of a single 5 mg zoledronic acid infusion in 25 patients with ISM and osteoporosis, showing clear biochemical and densitometric benefits within one year. After treatment, lumbar spine BMD increased by an average of 6.0 ± 4.4%, and total hip BMD rose by 2.4 ± 3.2%, both highly significant improvements. Bone turnover markers showed marked reductions: BSAP fell by roughly 1/3 at both 6 and 12 months, while serum C-terminal telopeptide decreased by 68% at 6 months and remained suppressed by 56% at 1 year. Notably, no new vertebral or non-vertebral fractures were recorded during FUP, despite the high baseline prevalence of skeletal fragility, and serum calcium, phosphorus, renal function, and tryptase remained stable throughout. Treatment tolerability was excellent, with only transient flu-like symptoms reported after infusion and largely prevented by prophylactic acetaminophen. Overall, these findings indicate that annual zoledronic acid provides a potent, durable suppression of bone turnover and meaningful BMD gains in ISM-related osteoporosis, offering fracture protection without the need for high-frequency dosing [63]. Another recently published case report further emphasizes the efficacy of zoledronic acid in SM-related bone disease, even when fractures occur in the presence of normal DXA findings, and underscores the importance of considering SM in the diagnostic work-up of unexplained skeletal fragility [64].

#### 5.2.2. Pamidronate in Monotherapy or in Combination with Interferon-α

Pamidronate (PAM) has emerged as one of the earliest and most effective therapeutic options for bone complications in SM, particularly in patients with severe osteoporosis and recurrent vertebral fractures. Case series have demonstrated that single intravenous infusions of PAM, typically at doses of 90–105 mg, lead to significant increases in lumbar spine BMD, with reported gains ranging from 16% to nearly 30% over FUP periods of 1 to 5 years. Patients often experience substantial and sustained relief of bone pain lasting several months after infusion, and repeated annual administration has been associated with long-term stabilization of fracture risk. Interestingly, the therapeutic effect appears more pronounced in trabecular bone of the spine compared with cortical bone of the femur, where bone density may remain unchanged or decline, underscoring site-specific responsiveness [65]. Importantly, PAM has been well tolerated in SM cohorts, with only transient post-infusion reactions such as mild pyrexia or musculoskeletal discomfort reported. Although data remain limited to case reports and small series, the consistent improvements in vertebral bone density and pain control highlight PAM as a valuable anti-resorptive agent in the management of SM-related osteoporosis, providing a foundation for subsequent exploration of other BPs and modern anti-resorptive therapies.

Early observations by Laroche et al. demonstrated that combining monthly pamidronate infusions (90 mg or 1 mg/kg) with low-dose interferon (INF)-α resulted in a robust and clinically meaningful increase in BMD far exceeding the response typically seen with BPs alone. Pamidronate and INF-α appear to act synergistically because each targets a different, complementary pathway driving skeletal fragility. Pamidronate provides rapid, potent suppression of MC-mediated osteoclast activation, markedly reducing bone resorption, while INF-α exerts a disease-modifying effect by directly inhibiting MC proliferation, decreasing MC burden, and lowering circulating tryptase [66]. In their 2007 cohort of four patients with extensive vertebral fragility, the 2-year IFN-pamidronate regimen produced a mean BMD gain of 16% at the spine and 5% at the femoral neck, accompanied by a 50% reduction in BTMs. These improvements were maintained during subsequent PAM monotherapy, and no new fractures occurred over 4 years [66]. The larger 2011 FUP study with 10 patients confirmed these results, showing an average annual spinal BMD increase of 12.6% and a 66% drop in CTX in those treated with the combination therapy, while PAM alone yielded only modest gains (≈2% annually at the spine) and no improvement at the hip. Importantly, across both cohorts, fracture risk was completely suppressed during treatment, and the combination regimen uniquely reduced serum tryptase, suggesting a direct effect on MC activity [67].

More recently, in a monocentric retrospective study of 37 patients with SM-related osteoporosis, mostly ISM (34/37 cases), the CEREMAST expert center evaluated the 1-year efficacy of several therapeutic regimens, including IFNα, PAM, IFN + PAM, masitinib, and midostaurin. Patients had a mean age of 53.5 ± 12.7 years, mean lumbar and hip T-scores of −2.4 ± 1.7 and −1.8 ± 0.8, respectively, and a high fracture burden: 81.1% had fragility fractures, and 96.6% of these were vertebral, with an average of 3.6 ± 3.1 vertebral fractures per patient. Treatment distribution included two IFN, 21 IFN + PAM, five PAM, two masitinib, and nine midostaurin (MIDO) patients. Only PAM alone and IFN + PAM produced significant improvements in BMD over 1 year, while IFN monotherapy and MIDO showed no clear benefit. Two patients on IFN + PAM sustained new fractures, but both had markedly high pre-treatment fracture counts (four and seven vertebral fractures). These findings support monthly PAM and combined IFN + PAM as effective short-term strategies for improving BMD in SM-related osteoporosis while highlighting the need for further studies on masitinib and newer tyrosine kinase inhibitors [68]. Together, these studies underscore the innovative therapeutic value of PAM, particularly when paired with IFNα. The consistent, substantial BMD gains and the generally low incidence of new fractures highlight this multimodal strategy as an effective approach for SM-related bone disease.

#### 5.2.3. Interferon-α in Monotherapy or in Combination

Early clinical evidence from multiple case reports strongly suggests that INF-α represents one of the earliest and most effective targeted strategies for severe osteoporosis associated with SM. Across reported cases, including three patients treated with INF α-2b for 6 months and a separate 33-year-old man with disabling vertebral osteoporosis refractory to clodronate, INF therapy consistently produced substantial clinical and biological improvements. Patients experienced resolution of severe back pain, significant gains in trabecular BMD (often after years of decline), and marked reductions in BM MC burden. In several cases, INF withdrawal led to relapse of pain, loss of BMD, and recurrence of MCs infiltration, underscoring its disease-modifying effect on MC proliferation and mediator release [69,70]. All case reports and studies regarding the employment of these therapeutic options in real life are summarized in Table 3.

### 5.3. Second Line: Denosumab

Denosumab is a fully human monoclonal IgG2 antibody that exerts potent anti-resorptive effects by selectively targeting RANKL [71]. This way, denosumab prevents RANK-RANKL interaction, thereby inhibiting osteoclast differentiation, activation, and survival. This mechanism mimics the physiological action of OPG. As a consequence, denosumab produces rapid, profound, and reversible suppression of bone resorption, leading to significant increases in BMD at both trabecular and cortical skeletal sites and a marked reduction in vertebral, non-vertebral, and hip fracture risk [72]. Unlike bisphosphonates, denosumab does not bind to bone matrix and does not accumulate in the skeleton; its anti-resorptive effect is strictly dependent on continued administration, with bone turnover rapidly rebounding upon discontinuation.

The safety and toxicity profile of denosumab is generally favorable. Hypocalcemia represents the most relevant acute metabolic adverse event, particularly in patients with vitamin D deficiency, impaired renal function, or high baseline bone turnover [73]. Therefore, adequate calcium and vitamin D repletion prior to initiation and during therapy is necessary. Infections, particularly of the skin and urinary tract, have been reported slightly more frequently than with placebo, reflecting the role of RANKL signaling in immune regulation [73]. Dermatologic reactions such as eczema and rash may occur but are usually mild. Rare but serious long-term complications include ONJ and AFFs [73]. Dental assessment before treatment initiation and avoidance of invasive dental procedures during therapy are recommended to mitigate ONJ risk.

A unique and clinically critical safety issue associated with denosumab is the occurrence of rebound-associated vertebral fractures following treatment discontinuation, attributed to rapid reactivation of osteoclast activity and transient overshoot in bone resorption [74]. This phenomenon underscores the necessity of treatment continuity or planned transition to an alternative anti-resorptive agent, typically a bisphosphonate, when denosumab is stopped.

#### Denosumab in Refractory or Bisphosphonate-Intolerant Patients and Current Studies in First Line

In 2015, Rossini et al. emphasized the potential of anti-RANKL therapy in SM-affected patients unable to tolerate BPs [75]. The first clinical evidence appeared with Orsolini et al., [76] who treated four postmenopausal ISM patients suffering from severe osteoporosis and intolerance to oral BPs. Denosumab 60 mg s.c. every 6 months led, after 12 months, to significant BMD increases at both lumbar and femoral sites, while BTMs resulted in complete suppression. Importantly, tryptase levels declined in all four patients, suggesting a potential secondary effect on MC activity beyond bone metabolism. No new fractures or adverse reactions occurred [76].

Additional clinical insight comes from Sanchez Lopez et al. [77] In 2018, they reported a 46-year-old woman with ISM, osteopenia, and high hip-fracture risk. The patient had a history of UP and GI symptoms (diarrhea, gastroesophageal reflux) treated with denosumab for 2 years after BPs were contraindicated in order to avoid further GI symptoms. Treatment improved bone status but was discontinued following the development of ONJ, showing a necrotic area approximately 2 cm in diameter, radiographically confirmed. Although the overall risk of ONJ with denosumab in osteoporosis populations remains low, estimated at approximately 1.4:1 compared with zoledronate in pooled clinical trials, this case highlights the need for dental vigilance. Collectively, this report emphasizes that rare but significant complications such as ONJ must be considered when selecting therapy for SM-related osteoporosis [77].

In 2025, Joven and Diemer reported one of the few long-term observations of denosumab in ISM-associated bone disease, describing a 38-year-old man with BM-proven ISM and low bone mass without prior fractures. After an initial course of three annual zoledronic acid 5 mg infusions, the patient showed modest lumbar spine improvements during the first 2 years (+3.1% and +5.3%), followed by a −4.1% decline after the 3rd dose, while hip BMD remained largely unchanged. Because of this suboptimal response, he was transitioned to denosumab 60 mg every 6 months, which he continued for 5 years, achieving remarkable cumulative gains of +24.6% in lumbar spine BMD and +39.9% at the total hip compared with baseline. Notably, despite these substantial skeletal responses, his serum tryptase remained elevated throughout and did not improve with either therapy. Over the entire observation period, the patient remained fracture-free [78].

Larger studies are needed to determine the true impact of denosumab on fracture prevention in SM. Abbas et al. [79] reported the case of a 78-year-old woman with a >50-year history of CM who later developed systemic symptoms, including GI discomfort, flushing, and temperature-induced skin changes. Initial evaluation revealed an elevated serum tryptase, a positive *cKITD816V* pathogenic variant, and markedly increased 24 h urinary prostaglandin D_2_, prompting a diagnostic BM biopsy that confirmed ISM. Despite long-term BP therapy since 2008, she sustained a low-trauma L4 compression fracture in 2019, with DXA in 2020 showing a lumbar spine T-score of −2.9 (BMD 0.819 g/cm^2^) and femoral neck T-score of −2.4 (BMD 0.697 g/cm^2^). Bisphosphonates were discontinued, and denosumab 60 mg every 6 months was initiated in March 2021. Over three years of therapy, she demonstrated a 7% increase in lumbar spine BMD, while femoral neck BMD remained stable. Serum tryptase levels, which had remained persistently elevated for years, declined from 28.7 ng/mL (2017) to 15.9 ng/mL (2020) before denosumab and subsequently fluctuated between 12.0 and 14.3 ng/mL during treatment, suggesting a possible reduction in MC burden. She experienced no new fractures and remained clinically stable throughout FUP. Unlike the abovementioned case report, this case strengthens the hypothesis that denosumab may exert a disease-modifying effect by reducing systemic MC activity [79].

In this context, the ongoing randomized controlled trial (RCT) NCT03401060 is evaluating the efficacy and safety of denosumab 60 mg administered s.c. every 6 months for 3 years, compared with placebo in patients with SM-related osteoporosis. The trial is based on the hypothesis that denosumab, by targeting RANKL, may provide superior improvements in lumbar spine BMD and reduce new skeletal events, aided by the drug’s short half-life and reversibility. Participants are randomized to receive either denosumab or a placebo solution following identical injection schedules. Although definitive conclusions await the trial’s results, denosumab currently represents the most biologically targeted anti-resorptive option available for SM-associated osteoporosis.

Studies and case reports regarding the employment of denosumab in SM patients are summarized in Table 4.

### 5.4. Tyrosine Kinase Inhibitors and Their Role in Treating SM-Related Bone Disease

Midostaurin, avapritinib, bezuclastinib, and elenestinib represent the current generation of KIT-directed TKIs developed for SM, each with distinct pharmacologic selectivity for the constitutively activated *cKITD816V* variant that drives MC proliferation and mediator release in the majority of patients (Table 5).

Midostaurin, the 1st TKI approved for AdvSM, inhibits multiple kinases, including *KIT*, *FLT3*, *PDGFR*, and *VEGFR*, producing clinically meaningful reductions in MC burden, serum tryptase, splenomegaly, and symptoms; however, its modest potency against *cKITD816V* and frequent GI toxicity have limited its use in less advanced disease [80].

Avapritinib, a highly selective type I inhibitor that binds the active conformation of *cKITD816V* with nanomolar affinity, has demonstrated superior cytoreduction and symptom improvement in trials such as EXPLORER [81] and PATHFINDER [82], leading to its approval for AdvSM due to rapid and deep reductions in BM MCs and tryptase levels, including frequent complete remissions of the AHN component.

In addition, in May 2023, the U.S. Food and Drug Administration (FDA) granted approval for avapritinib in adults with ISM, based on results of the randomized, double-blind, placebo-controlled PIONEER trial demonstrating significant and clinically meaningful reductions in symptom burden and MC biomarkers compared with placebo. Parallel regulatory decisions in Europe authorize avapritinib for ISM with moderate to severe symptoms inadequately controlled by conventional therapy, further underscoring its potential to shift therapeutic paradigms for both indolent and advanced forms of the disease [83,84].

Bezuclastinib (CGT9486) is a next-generation TKI designed to minimize off-target toxicity while preserving high potency against mutant KIT. Early data from the ongoing SUMMIT trial (NCT05186753) briefly comment on its efficacy and safety in non-advanced SM, where 100 mg daily is well tolerated and highly active, and the drug is also being evaluated in AdvSM within the APEX study (NCT04996875).

Elenestinib (BLU-263), another selective inhibitor engineered to retain potency against *cKITD816V* while avoiding some of the adverse effects associated with avapritinib, is currently under investigation in a large Phase 1/2 open-label trial (AZURE) assessing its role in advanced SM, SM-AHN, and other KIT-altered hematologic malignancies. This study aims to determine the recommended doses of elenestinib both as monotherapy and in combination with azacitidine, with primary endpoints including safety, tolerability, and efficacy, and incorporates an estimated 4-year participation duration (2 years of therapy followed by 2 years of follow-up).

Some of the above-mentioned KIT inhibitors have emerged as promising disease-modifying agents for SM-related bone involvement:

In the phase 2 PATHFINDER trial, serial DXA scans from 56 evaluable AdvSM patients demonstrated significant and sustained improvements in lumbar spine BMD over a median 22-month FUP. Patients with low baseline BMD (BDlow; n = 12) improved their mean lumbar spine T-score from −2.44 to −1.63 (*p* = 0.034), with 58% achieving ≥0.5 SD increases and 80% of osteoporotic patients showing meaningful gains, while normal and high-density cohorts maintained stable BMD [82,83]. Exploratory data from the randomized PIONEER trial in ISM showed similar biomarker reductions and stability or improvement in bone measures with low-dose avapritinib (though not powered for bone endpoints) [84].

Bezuclastinib (SUMMIT) has demonstrated reductions in symptom burden and MC biomarkers, but no DXA or fracture outcomes have yet been reported.

Apart from the AZURE study, elenestinib is being evaluated in an ongoing phase 2/3 study of patients with ISM (HARBOR), which prospectively incorporates DXA, BTMs, and vertebral imaging. Early results show reductions in MCs’ burden, but no mature BMD or fracture data are yet available. Mechanistically, *KITD816V* inhibition reduces MC-derived osteoclastogenic mediators—histamine, tryptase, IL-6, TNF-α, and RANKL—restoring the RANKL/OPG balance, decreasing osteoclastogenesis, and permitting osteoblast-driven bone accrual. Despite encouraging findings, limitations include the absence of fracture endpoints, potential confounding from concomitant osteoporosis therapies (BPs in 7%, vitamin D/calcium in 46%, corticosteroids in 59% of DXA-evaluable patients), and heterogeneity of bone phenotypes across SM subtypes.

Overall, avapritinib currently provides the clearest evidence of improving low BMD in SM [85,86], whereas the skeletal effects of bezuclastinib and elenestinib remain biologically plausible, but dedicated bone-endpoint data are still pending (Table 5).

### 5.5. Treatment of Bone Pain Caused by MC Infiltration and Degranulation

As anticipated in the epidemiology section, bone pain is a frequent and clinically burdensome manifestation of SM, affecting 36.1% of patients in one of the largest contemporary U.S. cohorts (119/330 cases) and occurring alongside skeletal involvement (osteolytic lesions, fractures, or sclerosis) [87]. Because bone pain in SM is multifactorial, ranging from MC-mediated inflammation to true structural bone disease, rigorous differential diagnosis is essential, as common unrelated conditions such as osteoarthritis, mechanical back pain, or fibromyalgia account for many referrals. When SM-driven, pain may arise from MCs’ degranulation, providing a rationale for symptomatic therapies such as H1/H2 antihistamines and oral or inhaled sodium cromoglicate, although evidence remains limited to isolated case reports [88]. Both denosumab and peg-INF alfa-2a are useful for patients with refractory bone pain in patients not responding to bisphosphonates or for those who are not candidates for bisphosphonates because of renal insufficiency [89]. Palliative radiotherapy is usually reserved for refractory, focal osteolytic lesions. Among disease-directed therapies, midostaurin has demonstrated meaningful improvement in bone pain and skeletal symptoms. In a Polish real-world study, data involving 13 patients with aggressive SM noted that 77% experienced clinical benefit, often by the second month, with relief of mediator-related symptoms, including bone pain, and among evaluable patients, midostaurin reduced serum tryptase by a median 74% and cut BM MC burden by 50% at 6 months [90]. Therefore, despite GI and hematologic toxicities, midostaurin remains a valuable option for AdvSM with skeletal involvement.

### 5.6. Focus on Dickkopf-1 and Sclerostin and the Potential Therapeutic Role of Sclerostin-Antagonists

Dickkopf-1 and sclerostin are pivotal negative regulators of the canonical Wnt/β-catenin pathway and therefore play an important role in the pathophysiology of osteoporosis by inhibiting osteoblast differentiation and activity, reducing bone formation, and indirectly favoring bone resorption. In the general population, increased circulating levels of these proteins are commonly associated with low BMD, impaired bone microarchitecture, and increased fracture risk. Cross-sectional studies in ISM have consistently shown that serum DKK1 levels are significantly elevated, approximately 2-fold compared with age- and sex-matched controls, and correlate positively with parathyroid hormone as well as BTMs, supporting the concept that DKK1 overexpression is largely a secondary adaptive response to accelerated bone remodeling rather than a primary cause of osteoblast suppression [91]. Therefore, DKK1 appears more suitable as a biomarker of high bone turnover than as a direct pharmacologic target.

Focusing on sclerostin, preclinical models confirmed that sclerostin deficiency or antibody-mediated neutralization robustly increases BMD by uncoupling bone formation from resorption [92]. More recently, in a multicenter study including 39 patients with mastocytosis, spanning indolent SM, aggressive SM, smoldering SM, cutaneous mastocytosis, and SM with associated hematologic neoplasm, circulating sclerostin and bioactive sclerostin levels were systematically evaluated alongside in vitro MC experiments [15]. Neoplastic human MCs (HMC-1.2, *KIT D816V+*) were shown to constitutively secrete sclerostin, with IL-6 stimulation (100 ng/mL) inducing a significant upregulation of SOST gene expression at 6 h and 24 h, accompanied by a near 2-fold increase in sclerostin protein secretion at 24 h. Clinically, plasma sclerostin concentrations were significantly higher in advanced-stage diseases, with median levels of 34.2 pmol/L in ASM, SM-AHN, and SSM versus 19.3 pmol/L in ISM and SM. Markedly elevated values were especially found in SM-AHN: 55.9 pmol/L. Higher sclerostin levels were also observed in patients with serum tryptase > 20 µg/L and splenomegaly. Low-dose bone CT analysis revealed significantly increased sclerostin concentrations in patients with trabecular bone sclerosis compared with those without sclerosis. Correlation analyses demonstrated a moderate inverse correlation with BSAP, supporting an inhibitory effect on osteoblastic activity [15].

Collectively, these data identify MCs as a previously unrecognized source of sclerostin and provide a mechanistic and clinical rationale for targeting the Wnt–sclerostin axis as a potential therapeutic strategy for SM-related bone disease. In this context, anti-sclerostin monoclonal antibodies have recently been developed. However, there are no current studies that can clarify the efficacy of these agents even in the SM setting. Among these relatively new drugs, romosozumab and blosozumab have proved to exert a potent pharmacologic antagonism, inducing a marked anabolic response with rapid BMD gains, transient stimulation of bone formation markers [such as P1NP (a circulating marker of bone formation) generated by the processing of type 1 collagen], and sustained suppression of bone resorption [93].

**Figure 4:**
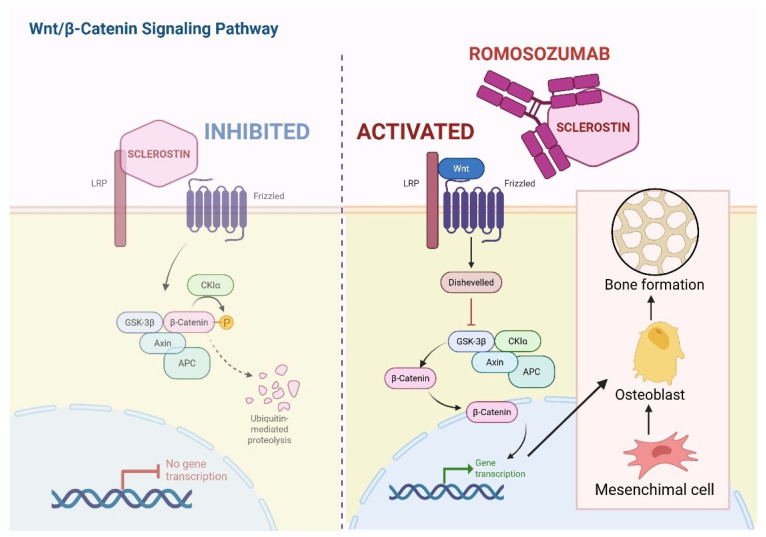
Mechanism of action of romosozumab, a monoclonal antibody that acts as a sclerostin inhibitor. LRP (low-density lipoprotein receptor protein 5 and 6); axin (a scaffold protein); APC (adenomatous polyposis coli-tumor suppressor protein); GSK3β (glycogen synthase kinase 3 beta); P (phosphate); CK1α (casein kinase 1 alpha); disheveled (cytoplasmic protein).

Focusing on romosozumab, it is a humanized IgG2 monoclonal antibody produced by recombinant DNA technology in Chinese hamster ovary cells, which inhibits sclerostin and activates the Wnt/β-Catenin pathway by preventing sclerostin-LRP5/6 interaction (Figure 4). Detailed analyses from the FRAME trial demonstrated that this drug induces a rapid and marked increase in bone formation within the first 2 months of treatment, with a 3–4-fold rise in mineralizing surface and bone formation rate in cancellous and endocortical bone, while simultaneously reducing bone resorption parameters by approximately 50%. By 12 months, bone formation indices decline, but suppression of bone resorption persists, leading to reduced overall bone turnover, increased trabecular thickness, improved trabecular connectivity, greater bone volume fraction, and higher tissue mineral density, thereby explaining the substantial gains in BMD and fracture risk reduction observed clinically [94]. Building on this mechanistic background, a recent 36-month prospective–retrospective study evaluated the addition of romosozumab to ongoing denosumab therapy in postmenopausal women who fractured or responded inadequately to long-term denosumab. In this cohort of 50 women, adding romosozumab to denosumab preserved the anabolic response, with a non-significant but favorable trend toward greater lumbar spine BMD gain compared with continued denosumab alone.

Importantly, ongoing RANKL inhibition did not blunt romosozumab-induced bone formation, suggesting that combined anti-resorptive and anabolic strategies may overcome therapeutic failure and mitigate rebound concerns after prolonged denosumab exposure [95]. Among potential side effects, romosozumab has been shown to be associated with cardiovascular toxicity [94]. Together, these studies support romosozumab as a mechanistically distinct agent capable of inducing rapid and sustained anti-resorptive effects, and they provide a strong rationale for its strategic use, alone or in combination with denosumab, in patients with severe or treatment-refractory osteoporosis.

### 5.7. Addressing the Other Side of the Coin: Osteoslerosis

The phase 2 PATHFINDER study provides compelling evidence that avapritinib exerts profound and sustained effects on bone disease in systemic mastocytosis by targeting the underlying neoplastic MC burden [96]. This also holds true for the osteosclerotic setting. Importantly, DXA changes were paralleled by marked histopathologic improvements, underscoring that osteosclerosis and myelofibrosis are direct consequences of high MC infiltration and activity. Across all BMD cohorts, myelofibrosis scores progressively shifted toward lower grades: the proportion of patients with scores 0–1 increased from 55% to 90% in BDlow, from 48% to 84% in BDhigh, and from 50% to 85% in BDnorm, while higher-grade fibrosis (scores 2–3) fell from 45% to 10% in BDlow patients. Similarly, osteosclerosis regressed substantially, with the proportion of patients scoring 0–1 rising from 80% to 100% in BDlow, from 60% to 73% in BDhigh, and from 59% to 93% in BDnorm within 40 weeks. These skeletal improvements closely tracked depletion of neoplastic MC infiltrates, with BM MC aggregates decreasing from 100% to 42% in BDlow, 95% to 14% in BDhigh, and 100% to 26% in BDnorm patients. Together, these findings demonstrate that osteosclerosis in SM is a manifestation of excessive MC burden and mediator-driven bone remodeling and that avapritinib, through potent and selective inhibition of *KITD816V*, can reverse fibrosis, reduce osteosclerosis, and restore more physiological bone structure. This represents the first robust evidence that targeted MCs eradication can directly translate into meaningful and durable improvements in skeletal pathology in SM [96].

## 6. Machine Learning and Osteoporosis: Current Evidence and Clinical Perspectives

Over the past decade, machine learning (ML) and deep learning (DL) approaches have transformed osteoporosis detection, risk stratification, and opportunistic screening. Contemporary ML-based frameworks leverage multimodal data (imaging, clinical variables, and body composition metrics) to improve diagnostic accuracy, scalability, and accessibility, particularly in aging populations at high fracture risk. Recently, Zhang et al. outlined the evolution from traditional ML models using structured clinical data to advanced DL systems incorporating high-dimensional imaging inputs from X-ray, CT, and MRI. While classical ML algorithms perform adequately with demographic and laboratory variables, DL models consistently demonstrate superior accuracy through automated feature extraction, especially for imaging-based bone quality assessment [97].

Importantly, ML-based osteoporosis detection aligns with broader AI-driven strategies aimed at fracture prevention and fall risk reduction in older adults. As highlighted in recent PRISMA-guided reviews of fall detection systems, osteoporosis is a central biological substrate linking skeletal fragility to fall-related morbidity and mortality [98]. Integrating ML-derived bone health metrics with wearable sensors, body composition analysis, and clinical risk factors could enable comprehensive, personalized fracture prevention pathways [98]. Collectively, the available evidence demonstrates that ML and DL technologies substantially enhance osteoporosis detection compared with traditional approaches and hold promise for scalable, personalized skeletal health monitoring and earlier intervention to reduce the global burden of osteoporotic fractures.

### How to Apply ML to the SM Setting in Order to Implement Bone Disease Management

Beyond population-level osteoporosis screening, ML may offer a particularly useful framework for individualized prediction of osteoporosis and fracture risk in SM, a setting characterized by heterogeneous mechanisms of bone disease that are not fully captured by DXA alone [99]. Unlike age-driven primary osteoporosis, SM-associated bone fragility often occurs at a younger age, may coexist with osteosclerosis, and is strongly influenced by MCs mediator release, clonal burden, and disease activity. ML models can integrate multidimensional data layers that are routinely fragmented in clinical practice, including demographic variables (younger age at bone involvement, male sex), mediator-related symptoms (recurrent anaphylaxis, flushing, diarrhea reflecting histamine, tryptase, and cytokine excess), BTMs (elevated PINP, CTX, TRAcP-5b indicating uncoupled bone remodeling), sclerostin levels, fracture history (particularly vertebral fragility fractures that may precede overt osteoporosis), *KIT D816V* VAF in PB or BM and molecular patterns of associated hematologic neoplasms (for example *S/A/R* mutations: *SRSF2*, *ASXL1*, *RUNX1*). Adverse molecular signatures (high *KIT D816V* VAF, marked BM MCs infiltration, persistently elevated serum tryptase, and pro-osteoclastogenic cytokine profiles) may be weighted alongside imaging-derived features from DXA or opportunistic CT (vertebral attenuation, volumetric trabecular metrics) to improve prediction not only of low bone density phenotypes and fracture risk, but also of high-turnover osteosclerotic patterns, in which increased BMD is paradoxically associated with skeletal fragility and bone pain. Importantly, ML models can be trained to account for and minimize confounding causal factors that influence bone metabolism in SM, such as chronic glucocorticoid exposure, vitamin D deficiency, hypogonadism, proton-pump inhibitor use, renal dysfunction, and concomitant anti-resorptive therapies, thereby isolating the SM-specific contribution to skeletal risk. By integrating clinical, biochemical, molecular, and imaging data over time, ML-driven prediction tools have the potential to identify high-risk SM patients early, guide timing of DXA [99] or CT-based surveillance, support decisions on anti-resorptive or anabolic therapy, and monitor skeletal response to disease-modifying treatments. In this context, ML does not merely replicate traditional osteoporosis screening but enables a disease-informed, precision-medicine approach to bone health in SM, where fracture risk may be underestimated by conventional algorithms and early intervention can substantially reduce morbidity [99].

## 7. Controversies, Unmet Needs, and Future Directions

Despite major advances in the understanding of MC-driven bone remodeling, the management of bone disease in SM remains characterized by some unresolved controversies and substantial unmet clinical needs:

- When should anti-osteoporotic therapy be initiated?

Based on current evidence, the decision to initiate BP therapy in SM should not rely on BMD thresholds alone but rather on an integrated assessment of fracture risk that reflects the unique pathophysiology of MC-driven bone disease. The presence of a fragility fracture (particularly vertebral fractures), whether clinical or morphometric, constitutes a clear and absolute indication for anti-resorptive therapy, irrespective of T-score or Z-score values, as fractures in SM frequently occur even in patients without densitometric osteoporosis. In the absence of documented fractures, BP therapy is generally recommended in patients with densitometric osteoporosis, defined as a T-score ≤ −2.5 at the lumbar spine or hip in postmenopausal women and men aged ≥50 years. However, because SM often affects younger individuals and men, Z-scores are particularly relevant: a Z-score ≤ −2.0, especially when associated with elevated BMTs, male sex, or additional SM-specific risk factors (such as high tryptase levels or absence of cutaneous involvement), should prompt strong consideration of treatment even in the absence of fractures. Patients with osteopenia (T-score between −1.0 and −2.5) may also merit therapy if they exhibit high-risk features, including rapid BMD loss, markedly increased bone resorption, multiple clinical risk factors, or a high estimated fracture risk. Importantly, isolated low BMD without evidence of increased turnover or clinical risk may justify close monitoring rather than immediate treatment.

- Z-score versus T-score in Systemic Mastocytosis

Although Z-scores are specifically designed to assess BMD relative to age- and sex-matched reference populations and are therefore recommended for premenopausal women, men under 50 years, and secondary osteoporosis, most clinical studies in SM continue to rely almost exclusively on T-scores. This practice represents a conceptual inconsistency, as SM-related bone disease frequently affects individuals who fall outside the demographic for which T-scores were originally intended. The persistent use of T-scores in SM likely reflects historical extrapolation from postmenopausal osteoporosis frameworks and the absence of disease-specific guidelines, rather than biological appropriateness. In line with what we expressed in the previous point, future studies should prioritize the validation and routine use of Z-scores in SM, as their integration is essential to accurately identify pathological bone involvement and to move beyond other osteoporosis paradigms that may inadequately reflect this disease [32].

- The Need for SM-Specific Fracture Risk Prediction Models

In parallel, the absence of a validated, disease-specific fracture risk model further complicates clinical decision-making in SM. Although the MastFx score represents an important first attempt to quantify fracture risk in ISM by integrating readily available clinical and biochemical variables, its derivation from a single-center cohort and the lack of external validation currently limit its generalizability. Moreover, the score was developed in a context where BMD, largely expressed as T-scores, remained a central component, potentially underestimating risk in younger patients or in those with preserved BMD. Furthermore, future risk models will need to move beyond conventional osteoporosis frameworks and incorporate dynamic disease-specific parameters, including BMTs, *KIT D816V* VAF, and clinical phenotypes. Prospective multicenter validation of MastFx and its possible refinement through modern analytical approaches represents a potential strategy for personalized fracture prevention in SM.

- Therapeutic gaps

From a therapeutic standpoint, a major unmet need is the paucity of comparative and prospective evidence to guide treatment decisions in SM-related bone disease. The available data supporting BPs and INF-α largely stem from case reports, small case series, or retrospective cohorts, while clinical experience with denosumab remains limited to a handful of observational reports and individual cases. Although pamidronate, particularly in combination with INF-α, and denosumab appear effective in selected high-risk patients, robust data defining optimal treatment sequencing, duration of therapy, and the safety of treatment discontinuation or drug holidays are lacking. Notably, despite the strong biological rationale for targeting the Wnt–sclerostin pathway in MC-driven bone disease, no clinical studies have evaluated anabolic agents such as romosozumab in patients with SM. As a result, the efficacy and safety of Wnt-modulating therapies in this setting remain entirely unexplored and warrant dedicated, disease-specific clinical investigation. Finally, these therapeutic uncertainties underscore the critical need for integrated, SM-informed risk stratification tools to support individualized treatment strategies.

## 8. Conclusions

Bone disease represents a recognized component of SM, contributing substantially to long-term morbidity. SM-related skeletal involvement is biologically complex, often manifests at a younger age, frequently affects men, and displays a striking dissociation between BMD and fracture occurrence. These features reflect the unique pathophysiology of MC-driven bone remodeling, in which constitutive KIT activation, mediator excess, and altered cytokine signaling converge to disrupt both osteoclastic and osteoblastic homeostasis. Unfortunately, many questions remain unresolved, and a final consensus on how to approach SM-bone disease is warranted. Over the past decade, major advances have reshaped the management of SM-associated bone disease. Traditional anti-resorptive strategies, particularly intravenous bisphosphonates, remain a cornerstone of therapy and provide meaningful fracture protection, especially in trabecular bone. The advent of highly selective KIT inhibitors has redefined therapeutic expectations. Particularly, avapritinib has provided the first robust evidence that targeted eradication of neoplastic MCs can translate into sustained improvements in BMD, regression of osteosclerosis, resolution of myelofibrosis, and depletion of BM MC infiltrates, firmly establishing bone pathology as a reversible manifestation of disease activity rather than a fixed complication. These findings support a paradigm shift in which control of the underlying clonal disorder becomes integral to skeletal management, particularly in advanced SM. Lastly, by integrating age, sex, mediator-related symptoms, bone turnover markers, molecular features such as *KIT D816V* allele burden and adverse mutational profiles, imaging-derived bone quality metrics, and confounding metabolic factors, ML-based models could overcome the limitations of DXA-centric algorithms and enable earlier, disease-informed intervention. Such approaches align naturally with the broader shift toward precision medicine and have the potential to transform skeletal surveillance, therapeutic decision-making, and outcome prediction in SM. Ultimately, only a truly integrated approach that unites clonal disease control, skeletal imaging, molecular risk profiling, and data-driven predictive models will allow SM-related bone disease to be managed proactively, leading to a marked improvement in patients’ quality of life.

## Figures and Tables

**Table 1:** Epidemiology of bone manifestations in systemic mastocytosis. ABBREVIATIONS: SM (systemic mastocytosis); No. (number); pts (patients); BMD (bone mineral density); REF (reference); ISM (indolent SM); AdvSM (adverse SM); BMM (bone marrow mastocytosis).

SM Subtype/ Patients Group	No. of pts/Study Type	Prevalence of Osteoporosis/Low BMD/Fractures	Important Notes	REF
ISM	157 ISM patients (65 men + 92 women) in the Dutch cohort	Osteoporosis: 28%; osteoporotic fractures: 37% (especially vertebral).	Higher prevalence in male individuals and older age.	Van der Veer E, (2012) The Netherlands [36]
ISM vs. AdvSM	61 patients (ISM n = 29, AdvSM n = 32)	Osteoporosis: 38% ISM 6% of AdvSM.	AdvSM is more likely to have increased BMD and osteosclerosis.	Riffel P, (2020) Austria [38]
Patients referred for osteoporosis (monocentric, retrospective study)	8392 patients with osteoporosis; 1374 had a bone biopsy	Osteoporosis: 0.5% had ISM; >5% young men with unexplained osteoporosis had ISM.	ISM is not trivial among osteoporosis patients, especially younger men.	Gehlen M, (2021) Germany [37]
ISM/BMM	431 SM cases (Verona region, Italy)	Osteoporosis: 35% of SM cases (fragility fractures are also frequent).	Includes BMM variant; affects younger males too.	Zanotti R, (2021) Italy [39]
Pooled Analysis	Pooled from the literature in the SM overall and the ISM subset	Osteoporosis ranges from 18 to 60 ISM, specifically, about 20–38%. Fragility fractures: ~30%.	Prevalence depends on diagnostic criteria, patient age and sex, and vertebral morphometry.	Jankovski L, (2025) Slovenia [35]

**Table 2:** Clinical, laboratory, and radiologic approaches for the diagnosis and monitoring of SM-related bone disease. ABBREVIATIONS: SM (systemic mastocytosis); RX (radiographs); CT (computer tomography); HU (Hounsfield units); BM (bone marrow); MC (mast cells); AdvSM (adverse systemic mastocytosis); MRI (magnetic resonance imaging); PET-CT (positron emission tomography-CT); ISM/SSM (indolent/smoldering SM); AHN (associated hematologic neoplasm); MCS (mast cell sarcoma).

Radiological Approaches	What to Assess?	Rationale in SM	Notes
Vertebral Imaging (RX)	Baseline thoraco-lumbar X-ray.	~30% vertebral fractures are silent. X-rays detect: Deformities; Cortical thinning; Sclerotic lesions.	Height loss (≥2 cm recent or ≥4 cm historical) triggers imaging.
Radiography (General Skeletal Survey)	Focal/multifocal/diffuse osteosclerosis. Lytic lesions (with sclerotic rim).	Limited sensitivity osteoporosis is not visible until ~30% bone loss. [Important for suggesting SM when integrated with clinical data].	Sclerotic Findings may mimic metastases osteopoikilosis [55].
CT	Small lesions. Complex anatomy (pelvis, spine). Cortical thickening. HU (BM attenuation).	Detects focal lesions. ↑ HU values ↑ MC burden (AdvSM).	Low-dose whole-body CT becomes useful when radiographs are inconclusive [55].
MRI/Whole-Body MRI	Marrow infiltration. Distinguishing patterns: - Activated marrow; (T1 ↓, T2/TIRM ↑). - Sclerotic marrow (T1 ↓, T2 ↓). Lytic lesions (T1 ↓, T2 ↑).	- Most sensitive modality for BM disease. - Identifies MC-related BM patterns. - Partial value in treatment response (T1 ↑, T2 ↓).	Not yet validated as a routine monitoring tool [55].
Scintigraphy/PET-CT	Diffuse vs. focal uptake.	Scintigraphy: Diffuse uptake reflects BM expansion (non-specific). PET-CT: Negative ISM/SSM/AdvSM. Positive SM-AHN or MCS.	- Limited role in routine. - Assesses BM involvement.

The downward arrow indicates a reduction, the upward arrow an increase.

**Table 3:** Case reports and studies describing the employment of bisphosphonates, interferon-α, and combinations of these 2 drugs in patients affected by SM-related osteoporosis. ABBREVIATIONS: BMD (bone mineral density); pts (patients); BTMs (bone turnover markers); BSAP (bone-specific alkaline phosphatase); CTX (C-terminal telopeptide); sT (serum tryptase); PAM (pamidronate); INF-α (interferon-α); frxs (fractures); MIDO (midostaurin).

Source	Population	Clinical Features	Therapy	Outcome	Additional Notes
Mathew 2009 [62]	36-year-old man	- Back pain - Osteoporosis - Multiple fractures	- Zolendronic acid 4 mg monthly - Prednisone 50 mg/day - Kyphoplasty	BMD stabilization ↓ Pain	Anaphylaxis during kyphoplasty Triggered by Acetaminophen
Rossini 2014 [63]	25 pts	- ISM - Osteoporosis - vertebral deformities (13/25) - mean lumbar T-score (−2.7)	- Zolendronate 5 mg single infusion	↑ Lumbar BMD by ~6.0 ± 4.4%, ↑ Hip BMD ~2.4 ± 3.2% ↓ BMTs: BSAP fell by 1/3 at 6 months CTX fell by 68% at 6 months - No new fractures	Transient flu-like symptoms
Parker 2024 [64]	48-year-old man	- Normal BMD - Femoral fracture - sT 171 ng/mL - ↑ BMTs - Osteosclerosis	Yearly zoledronic acid	↓ BMTs BMD gain: −15.8% spine −13% hip	Anaphylaxis during hip surgery Triggered by oxycodone
Laroche 2006 [66]	4 pts	- Vertebral fragility	PAM 90 mg/1 mg/kg +Low-dose INF-α for 2 years + PAM monotherapy Maintenance	BMD gain: −16% spine −5% hip - BMTs: 50% reduction	No new fractures over 4 years
Laroche 2011 [67]	10 pts	- Mean vertebral frxs: ~3.5 - Spinal T-score: −3 ± 1 - Hip T-score: −1.9 ± 0.7 - CTX: 357 ± 258 pg/mL - BSAP20 ± 3.2 IU - sT 49 ± 36 μg/mL	PAM 90 mg/1 mg/kg +Low-dose INF-α for 2 years 2 patients treated with PAM monotherapy	Combination therapy - BMD increase: 12.6% annually - CTX dropped by 66% - BSAP dropped by 25% - sT dropped by 34% PAM monotherapy BMD increase: −2.4 ± 0.1% spine −0 ± 01% hip	- Fracture risk was suppressed during treatment - The combination regimen uniquely reduced sT potential effect on MC activity
Nezzar 2023 [68]	37 pts	- ISM 34/37	- 2 IFNα - 5 PAM - 21 IFN + PAM, - 2 Masitinib - 9 MIDO	PAM and IFN + PAM: BMD improved over 1 year IFN and MIDO: no clear benefit	2 patients on IFN + PAM sustained new fractures, but both had high pre-treatment fracture counts (4 and 7 vertebral fractures)

The downward arrow indicates a reduction, the upward arrow an increase.

**Table 4:** Case reports, prior and ongoing studies on denosumab in the SM setting. ABBREVIATIONS: ISM (indolent systemic mastocytosis); pts (patients); BMD (bone mineral density); BTM (bone turnover markers); sT (serum tryptase); MCs (mast cells); ONJ (osteonecrosis of the jaw); CM (cutaneous mastocytosis); BPs (bisphosphonates); RCT (randomized clinical trial); ASM (aggressive SM); AHN (associated hematologic neoplasm); RANKL (Receptor Activator of Nuclear Factor kappa-B Ligand).

Source	Population	Clinical Features	Therapy	Outcome	Additional Notes
Orsolini 2015 [76]	4 pts All women	- Postmenopausal - ISM - Age 55–84 years - Lumbar T-scores −2.5 to −3.6, - Femoral T-scores −0.6 to −3.0 - Intolerant to BPs	60 mg s.c. every 6 months	After 1 year: ↑ BMD both spine and hip BTMs: undetectable levels	sT levels (baseline 19.6–35.4 µg/L), declined in all 4 patients a potential secondary effect on MC activity No new fractures No adverse reactions
Sànchez Lòpez 2018 [77]	46 y-old Woman	- ISM - Osteopenia - High fracture risk	60 mg every 6 months for 2 years	↑ BMD early suspension due to ONJ	Dental vigilance is mandatory, despite the fact that the ONJ risk remains low
Joven and Diemer 2025 [78]	38 y-old Man	Z-scores: - Spine −2.8 - Hip −2.5 No prior fractures	60 mg every 6 months (continued for 5 years) Prior therapy: - Zoledronic acid 5 mg 3 infusions/year suboptimal response	↑BMD: +24.6% lumbar spine +39.9% hip	No tryptase reduction No fractures occurred
Abbas 2025 [79]	78 y-old woman	>50 y history of CM Systemic symptoms Evolution to ISM	60 mg every 6 months from March 2021 BPs from 2008 to 2021 suboptimal response	Over 3 years: - ↑ 7% BMD spine - BMD hip stable	- sT levels fell to 12.0–14.3 ng/mL - No fractures - Possible disease-modifying effect
NCT03401060 RCT Denosumab vs. placebo	Patients with SM-related osteoporosis	INCLUSION CRITERIA - ≥18 years - ISM or CM - Spine T-score ≤ −2.5) - Osteopenia (T-score > −2.5 < −1.0) + history of low-energy fractures EXCLUSION CRITERIA - ASM or SM-AHN - Metabolic causes - Prior denosumab - Renal impairment - Pregnancy	Denosumab OR Placebo	Last update: 3 May 2024	RATIONALE: Denosumab, by targeting RANKL, may provide superior improvements in lumbar spine BMD and reduce new skeletal events

The downward arrow indicates a reduction, the upward arrow an increase.

**Table 5:** Tyrosine kinase inhibitors (TKIs) are currently employed or studied in the SM setting. This table summarizes the major TKIs approved in clinical practice for SM and those currently evaluated in clinical trials, their effect on the tyrosine kinase family, their current therapeutic indication in Italy, and their effect on SM-related bone disease. ABBREVIATIONS: TKI (tyrosine kinase inhibitor); VEGFR (vascular endothelial growth factor receptor); ISM/SSM/AdvSM (indolent/smoldering/advanced systemic mastocytosis); BMD (bone mineral density); BM (bone marrow).

TKIs	*cKITD816V*	*PDGFRα*	*PDGFRβ*	*FLT3*	VEGFR	SRC Family	Therapeutic Indication in Italy	Effect on Bone
Midostaurin	++	+	+	++++	+	+	≥1st LINE in AdvSM	↓ Bone pain (symptomatic)
Avapritinib BLU-285	++++	++++ (*D842V*)	+	_	_	±	1st LINE In ISM/SSM (when symptoms are not controlled) ≥2nd in AdvSM	↑ BMD ↓ sclerosis ↓ BM fibrosis - Symptomatic - Disease-modifying
Bezuclastinib CGT9486	++++	±	±	_	_	_	Experimental ISM/SSM (SUMMIT) AdvSM (APEX)	Trials ongoing
Elenestinib BLU-263	++++	±	±	_	_	_	Experimental ISM (HARBOR) AdvSM (AZURE)	Trials ongoing

The number of ‘+’ signs indicates the greater effect exerted on the target. The downward arrow indicates a reduction, the upward arrow an increase.

## Data Availability

No new data were created or analyzed in this study. Data sharing is not applicable to this article.

## Abbreviations

The following abbreviations are used in this manuscript:

AHN	Associated Hematologic Neoplasm
AFF	Atypical Femoral Fracture
AdvSM	Advanced Systemic Mastocytosis
AML	Acute Myeloid Leukemia
ASM	Aggressive Systemic Mastocytosis
B-Findings	Markers of High Mast Cell Burden
BMD	Bone Mineral Density
BM	Bone Marrow
MRI	Magnetic Resonance Imaging
BMM	Bone Marrow Mastocytosis
BMP	Bone Morphogenetic Protein
BMTs	Bone Turnover Markers
BSAP	Bone-Specific Alkaline Phosphatase
BTK	Bruton Tyrosine Kinase
C-Findings	Indicators of Organ Damage Requiring Cytoreduction
c-KIT/KIT	Tyrosine-Protein Kinase KIT
*cKITD816V*	KIT Aspartate 816 to Valine Mutation
CM	Cutaneous Mastocytosis
CMML	Chronic Myelomonocytic Leukemia
CTX	C-Terminal Telopeptide of Type I Collagen
CT	Computed Tomography
DXA	Dual-Energy X-ray Absorptiometry
ECTS	European Calcified Tissue Society
FDG-PET/CT	Fluorodeoxyglucose Positron Emission Tomography/Computed Tomography
FUP	Follow-Up
GI	Gastrointestinal
HIF-1α	Hypoxia-Inducible Factor 1 Alpha
IFN-α	Interferon Alpha
IL-11	Interleukin 11
ISM	Indolent Systemic Mastocytosis
KITM541L	KIT Methionine-to-Leucine Substitution at Codon 541
LRP5	Low-Density Lipoprotein Receptor–Related Protein 5
MCAS	Mast Cell Activation Syndrome
MCs	Mast Cells
MCL	Mast Cell Leukemia
MCS	Mast Cell Sarcoma
ML	Machine Learning
MSC	Mesenchymal Stem Cell
NGS	Next-Generation Sequencing
ONJ	Osteonecrosis of the Jaw
OPG	Osteoprotegerin
OS	Overall Survival
PB	Peripheral Blood
PDGFR	Platelet-Derived Growth Factor Receptor
QoL	Quality of Life
RANK	Receptor Activator of Nuclear Factor κB
RANKL	Receptor Activator of Nuclear Factor κB Ligand
sBT	Baseline Serum Tryptase
SFRP	Secreted Frizzled-Related Protein
SM	Systemic Mastocytosis
SM-AHN	Systemic Mastocytosis with Associated Hematologic Neoplasm
SM	Smoldering Systemic Mastocytosis
TGF-β	Transforming Growth Factor Beta
TKI	Tyrosine Kinase Inhibitor
VAF	Variant Allele Frequency
VEGFR	Vascular Endothelial Growth Factor Receptor
WB-MRI	Whole-Body MRI
Wnt	Wingless Signaling Pathway
WHO	World Health Organization

## References

[1] Khoury J.D., Solary E., Abla O., Akkari Y., Alaggio R., Apperley J.F., Bejar R., Berti E., Busque L., Chan J.K.C. (2022). The 5th Edition of the World Health Organization Classification of Haematolymphoid Tumours: Myeloid and Histiocytic/Dendritic Neoplasms. Leukemia.

[2] Fazio M., Vetro C., Markovic U., Duminuco A., Parisi M., Maugeri C., Mauro E., Parrinello N.L., Stagno F., Villari L. (2023). A Case of High-risk AML in a Patient with Advanced Systemic Mastocytosis. Clin. Case Rep..

[3] OROSCO TTAMINA A.L., Munoz J., Kelemen K., Palmer J., Al-Kali A., Rivera C., Gangat N., Pardanani A., Tefferi A., Arana Yi C. (2025). Systemic Mastocytosis with Associated Lymphoid Neoplasms (SM-ALN): A Distinct Subset with Indolent Clinical Course and Favorable Outcomes. Blood.

[4] Fazio M., Elena C., Ferrari J., Camilotto V., Stella S., Leotta D., Palumbo G.A., Di Raimondo F., Romano A. (2025). Is Bruton Tyrosine Kinase a Potential Target to Treat Mast Cell Neoplasms? Systemic Mastocytosis Associated with Chronic Lymphoid Leukemia Successfully Treated with Acalabrutinib Monotherapy: A Case Report and Review of the Literature. Haematologica.

[5] Rocha J., Luz Duarte M., Marques H., Torres F., Tavares P., Silva A., Brito C. (2010). Association of Adult Mastocytosis with M541L in the Transmembrane Domain of KIT. J. Eur. Acad. Dermatol. Venereol..

[6] Aldama L.N.D., Karlins E., Sun X., Veltri D., Komarow H.D., Maric I., Metcalfe D.D., Carter M.C. (2024). Prevalence and Impact of the *KIT* M541L Variant in Patients with Mastocytosis. Oncotarget.

[7] Banovac K., Renfree K., Makowski A.L., Latta L.L., Altman R.D. (1995). Fracture Healing and Mast Cells. J. Orthop. Trauma.

[8] Corrado A., Neve A., Macchiarola A., Gaudio A., Marucci A., Cantatore F.P. (2013). RANKL/OPG Ratio and DKK-1 Expression in Primary Osteoblastic Cultures from Osteoarthritic and Osteoporotic Subjects. J. Rheumatol..

[9] Ng C.W., Chan B.C.L., Ko C.H., Tam I.Y.S., Sam S.W., Lau C.B.S., Leung P.C., Lau H.Y.A. (2022). Human Mast Cells Induce Osteoclastogenesis through Cell Surface RANKL. Inflamm. Res..

[10] Rossini M., Zanotti R., Viapiana O., Tripi G., Orsolini G., Idolazzi L., Bonadonna P., Schena D., Escribano L., Adami S. (2014). Bone Involvement and Osteoporosis in Mastocytosis. Immunol. Allergy Clin. N. Am..

[11] Liu J., Chen X., Yu X. (2025). Unraveling the Role of N6-Methylation Modification: From Bone Biology to Osteoporosis. Int. J. Med. Sci..

[12] Sangadala S., Kim C.H., Fernandes L.M., Makkar P., Beck G.R., Boden S.D., Drissi H., Presciutti S.M. (2023). Sclerostin Small-Molecule Inhibitors Promote Osteogenesis by Activating Canonical Wnt and BMP Pathways. eLife.

[13] Letizia Mauro G., Accomando J., Tomasello S., Duca A., Mangano M.S., de Sire A., Vecchio M., Scaturro D. (2024). Osteoporosis in Systemic Mastocytosis: A Scoping Review. Medicina.

[14] Rabenhorst A., Christopeit B., Leja S., Gerbaulet A., Kleiner S., Förster A., Raap U., Wickenhauser C., Hartmann K. (2013). Serum Levels of Bone Cytokines Are Increased in Indolent Systemic Mastocytosis Associated with Osteopenia or Osteoporosis. J. Allergy Clin. Immunol..

[15] Szudy-Szczyrek A., Mlak R., Pigoń-Zając D., Krupski W., Mazurek M., Tomczak A., Chromik K., Górska A., Koźlik P., Juda A. (2025). Role of Sclerostin in Mastocytosis Bone Disease. Sci. Rep..

[16] Ragipoglu D., Dudeck A., Haffner-Luntzer M., Voss M., Kroner J., Ignatius A., Fischer V. (2020). The Role of Mast Cells in Bone Metabolism and Bone Disorders. Front. Immunol..

[17] Krstic J., Santibanez J.F. (2014). Transforming Growth Factor-Beta and Matrix Metalloproteinases: Functional Interactions in Tumor Stroma-Infiltrating Myeloid Cells. Sci. World J..

[18] Norrby K. (2024). On Connective Tissue Mast Cells as Protectors of Life, Reproduction, and Progeny. Int. J. Mol. Sci..

[19] Tobío A., Bandara G., Morris D.A., Kim D.-K., O’Connell M.P., Komarow H.D., Carter M.C., Smrz D., Metcalfe D.D., Olivera A. (2020). Oncogenic D816V-KIT Signaling in Mast Cells Causes Persistent IL-6 Production. Haematologica.

[20] Fischer V., Ragipoglu D., Diedrich J., Steppe L., Dudeck A., Schütze K., Kalbitz M., Gebhard F., Haffner-Luntzer M., Ignatius A. (2020). Mast Cells Trigger Disturbed Bone Healing in Osteoporotic Mice. J. Bone Miner. Res..

[21] Kim D.-K., Bandara G., Cho Y.-E., Komarow H.D., Donahue D.R., Karim B., Baek M.-C., Kim H.M., Metcalfe D.D., Olivera A. (2021). Mastocytosis-Derived Extracellular Vesicles Deliver MiR-23a and MiR-30a into Pre-Osteoblasts and Prevent Osteoblastogenesis and Bone Formation. Nat. Commun..

[22] Fazio M., Stagno F., Penna G., Mirabile G., Allegra A. (2025). Navigating the Landscape of Exosomal MicroRNAs: Charting Their Pivotal Role as Biomarkers in Hematological Malignancies. Non-Coding RNA.

[23] Wang M., Seibel M.J. (2023). Skin and Bones: Systemic Mastocytosis and Bone. Endocrinol. Diabetes Metab. Case Rep..

[24] Nakayama S., Yokote T., Hiraoka N., Akioka T., Nishiwaki U., Miyoshi T., Iwaki K., Fumimoto A., Masuda Y., Hatooka J. (2017). Transforming Growth Factor β—And Interleukin 13–Producing Mast Cells Are Associated with Fibrosis in Bone Marrow. Hum. Pathol..

[25] Wu M., Chen G., Li Y.-P. (2016). TGF-β and BMP Signaling in Osteoblast, Skeletal Development, and Bone Formation, Homeostasis and Disease. Bone Res..

[26] Han Y., Gao H., Gan X., Liu J., Bao C., He C. (2024). Roles of IL-11 in the Regulation of Bone Metabolism. Front. Endocrinol..

[27] You J., Liu M., Li M., Zhai S., Quni S., Zhang L., Liu X., Jia K., Zhang Y., Zhou Y. (2023). The Role of HIF-1α in Bone Regeneration: A New Direction and Challenge in Bone Tissue Engineering. Int. J. Mol. Sci..

[28] Komi D.E.A., Khomtchouk K., Santa Maria P.L. (2020). A Review of the Contribution of Mast Cells in Wound Healing: Involved Molecular and Cellular Mechanisms. Clin. Rev. Allergy Immunol..

[29] Ehnert S., Relja B., Schmidt-Bleek K., Fischer V., Ignatius A., Linnemann C., Rinderknecht H., Huber-Lang M., Kalbitz M., Histing T. (2021). Effects of Immune Cells on Mesenchymal Stem Cells during Fracture Healing. World J. Stem Cells.

[30] Rama T.A., Henriques A.F., Matito A., Jara-Acevedo M., Caldas C., Mayado A., Muñoz-González J.I., Moreira A., Cavaleiro-Rufo J., García-Montero A. (2023). Bone and Cytokine Markers Associated with Bone Disease in Systemic Mastocytosis. J. Allergy Clin. Immunol. Pract..

[31] Lobefalo L., Bali N., Lolli A. (2025). Calcium ion signals are fundamental in viral infection. Int. J. Infect..

[32] Avivar-Valderas A. (2023). Inhibition of PI3Kβ and mtor influence the immune response and the defense mechanism against pathogens. Int. J. Infect..

[33] Cohen S.S., Skovbo S., Vestergaard H., Kristensen T., Møller M., Bindslev-Jensen C., Fryzek J.P., Broesby-Olsen S. (2014). Epidemiology of Systemic Mastocytosis in Denmark. Br. J. Haematol..

[34] Meyer H.-J., Pönisch W., Monecke A., Gundermann P., Surov A. (2021). Bone Mineral Density in Patients with Systemic Mastocytosis: Correlations with Clinical and Histopathological Features. Clin. Exp. Rheumatol..

[35] Jankovski L., Rakusa M., Koceva A., Janež A., Kopač P., Jensterle M. (2025). Indolent Mastocytosis and Bone Health: Molecular Mechanisms and Emerging Treatment Options. Int. J. Mol. Sci..

[36] van der Veer E., van der Goot W., de Monchy J.G.R., Kluin-Nelemans H.C., van Doormaal J.J. (2012). High Prevalence of Fractures and Osteoporosis in Patients with Indolent Systemic Mastocytosis. Allergy.

[37] Gehlen M., Schmidt N., Pfeifer M., Balasingam S., Schwarz-Eywill M., Maier A., Werner M., Siggelkow H. (2021). Osteoporosis Caused by Systemic Mastocytosis: Prevalence in a Cohort of 8392 Patients with Osteoporosis. Calcif. Tissue Int..

[38] Riffel P., Schwaab J., Lutz C., Naumann N., Metzgeroth G., Fabarius A., Schoenberg S.O., Hofmann W.-K., Valent P., Reiter A. (2020). An Increased Bone Mineral Density Is an Adverse Prognostic Factor in Patients with Systemic Mastocytosis. J. Cancer Res. Clin. Oncol..

[39] Zanotti R., Bonifacio M., Isolan C., Tanasi I., Crosera L., Olivieri F., Orsolini G., Schena D., Bonadonna P. (2021). A Multidisciplinary Diagnostic Approach Reveals a Higher Prevalence of Indolent Systemic Mastocytosis: 15-Years’ Experience of the GISM Network. Cancers.

[40] Zanotti R., Tanasi I., Bernardelli A., Orsolini G., Bonadonna P. (2021). Bone Marrow Mastocytosis: A Diagnostic Challenge. J. Clin. Med..

[41] Zanotti R., Bonifacio M., Lucchini G., Sperr W.R., Scaffidi L., van Anrooij B., Oude Elberink H.N., Rossignol J., Hermine O., Gorska A. (2022). Refined Diagnostic Criteria for Bone Marrow Mastocytosis: A Proposal of the European Competence Network on Mastocytosis. Leukemia.

[42] Degboé Y., Eischen M., Apoil P.A., Mailhol C., Dubreuil P., Hermine O., Paul C., Bulai Livideanu C., Laroche M. (2019). Higher Prevalence of Vertebral Fractures in Systemic Mastocytosis, but Not in Cutaneous Mastocytosis and Idiopathic Mast Cell Activation Syndrome. Osteoporos. Int..

[43] Degboé Y., Severino-Freire M., Couture G., Apoil P.-A., Gaudenzio N., Hermine O., Ruyssen-Witrand A., Paul C., Laroche M., Constantin A. (2024). The Prevalence of Osteoporosis Is Low in Adult Cutaneous Mastocytosis Patients. J. Allergy Clin. Immunol. Pract..

[44] Özdemir Ö., Kasımoğlu G., Bak A., Sütlüoğlu H., Savaşan S. (2024). Mast Cell Activation Syndrome: An up-to-Date Review of Literature. World J. Clin. Pediatr..

[45] Hermine O., Lortholary O., Leventhal P.S., Catteau A., Soppelsa F., Baude C., Cohen-Akenine A., Palmérini F., Hanssens K., Yang Y. (2008). Case-Control Cohort Study of Patients’ Perceptions of Disability in Mastocytosis. PLoS ONE.

[46] Williams C., Anastasopoulou C., Sapra A. (2025). Biochemical Markers of Osteoporosis.

[47] Sperr W.R., Kundi M., Alvarez-Twose I., van Anrooij B., Oude Elberink J.N.G., Gorska A., Niedoszytko M., Gleixner K.V., Hadzijusufovic E., Zanotti R. (2019). International Prognostic Scoring System for Mastocytosis (IPSM): A Retrospective Cohort Study. Lancet Haematol..

[48] Muñoz-González J.I., Álvarez-Twose I., Jara-Acevedo M., Zanotti R., Perkins C., Jawhar M., Sperr W.R., Shoumariyeh K., Schwaab J., Greiner G. (2021). Proposed Global Prognostic Score for Systemic Mastocytosis: A Retrospective Prognostic Modelling Study. Lancet Haematol..

[49] Guillaume N., Desoutter J., Chandesris O., Merlusca L., Henry I., Georgin-Lavialle S., Barete S., Hirsch I., Bouredji D., Royer B. (2013). Bone Complications of Mastocytosis: A Link between Clinical and Biological Characteristics. Am. J. Med..

[50] Rossini M., Zanotti R., Bonadonna P., Artuso A., Caruso B., Schena D., Vecchiato D., Bonifacio M., Viapiana O., Gatti D. (2011). Bone Mineral Density, Bone Turnover Markers and Fractures in Patients with Indolent Systemic Mastocytosis. Bone.

[51] National Clinical Guideline Centre (2012). Osteoporosis: Fragility Fracture Risk: Osteoporosis: Assessing the Risk of Fragility Fracture.

[52] van der Veer E., Arends S., van der Hoek S., Versluijs J.B., de Monchy J.G.R., Oude Elberink J.N.G., van Doormaal J.J. (2014). Predictors of New Fragility Fractures after Diagnosis of Indolent Systemic Mastocytosis. J. Allergy Clin. Immunol..

[53] Rossini M., Adami S., Bertoldo F., Diacinti D., Gatti D., Giannini S., Giusti A., Malavolta N., Minisola S., Osella G. (2016). Guidelines for the Diagnosis, Prevention and Management of Osteoporosis. Reumatismo.

[54] Leone A., Macagnino S., D’ambra G., Perla D. (2021). Systemic Mastocytosis: Radiological Point of View. Mediterr. J. Hematol. Infect. Dis..

[55] Leone A., Criscuolo M., Gullì C., Petrosino A., Carlo Bianco N., Colosimo C. (2021). Systemic Mastocytosis Revisited with an Emphasis on Skeletal Manifestations. Radiol. Med..

[56] Murdaca G., Allegra A., Tonacci A., Musolino C., Ricciardi L., Gangemi S. (2022). Mast Cells and Vitamin D Status: A Clinical and Biological Link in the Onset of Allergy and Bone Diseases. Biomedicines.

[57] Kim B., Cho Y., Lim W. (2021). Osteoporosis Therapies and Their Mechanisms of Action (Review). Exp. Ther. Med..

[58] Dömötör Z.R., Vörhendi N., Hanák L., Hegyi P., Kiss S., Csiki E., Szakó L., Párniczky A., Erőss B. (2020). Oral Treatment with Bisphosphonates of Osteoporosis Does Not Increase the Risk of Severe Gastrointestinal Side Effects: A Meta-Analysis of Randomized Controlled Trials. Front. Endocrinol..

[59] Pazianas M., Abrahamsen B. (2011). Safety of Bisphosphonates. Bone.

[60] Robinson D.E., Ali M.S., Pallares N., Tebé C., Elhussein L., Abrahamsen B., Arden N.K., Ben-Shlomo Y., Caskey F.J., Cooper C. (2020). Safety of Oral Bisphosphonates in Moderate-to-Severe Chronic Kidney Disease: A Binational Cohort Analysis. J. Bone Miner. Res..

[61] Curtis E.M., Moon R.J., Dennison E.M., Harvey N.C., Cooper C. (2016). Recent Advances in the Pathogenesis and Treatment of Osteoporosis. Clin. Med..

[62] Mathew R., Dhillon V., Shepherd P. (2009). Systemic Mastocytosis Presenting as Osteoporosis—A Case Report. Clin. Rheumatol..

[63] Rossini M., Zanotti R., Viapiana O., Tripi G., Idolazzi L., Biondan M., Orsolini G., Bonadonna P., Adami S., Gatti D. (2014). Zoledronic Acid in Osteoporosis Secondary to Mastocytosis. Am. J. Med..

[64] Parker J.A., Hou R. (2024). A 48-Year-Old Man with a Hip Fracture and Skin Rash: A Case Report. AACE Clin. Case Rep..

[65] Marshall A., Kavanagh R.T., Crisp A.J. (1997). The Effect of Pamidronate on Lumbar Spine Bone Density and Pain in Osteoporosis Secondary to Systemic Mastocytosis. Rheumatology.

[66] Laroche M., Bret J., Brouchet A., Mazières B. (2006). Clinical and Densitometric Efficacy of the Association of Interferon Alpha and Pamidronate in the Treatment of Osteoporosis in Patients with Systemic Mastocytosis. Clin. Rheumatol..

[67] Laroche M., Livideanu C., Paul C., Cantagrel A. (2011). Interferon Alpha and Pamidronate in Osteoporosis with Fracture Secondary to Mastocytosis. Am. J. Med..

[68] Nezzar C., Alary P., Ruyssen-Witrand A., Couture G., Severino-Freire M., Laroche M., Constantin A., Bulai Livideanu C., Degboe Y. (2023). Pos0496 Management of Osteoporosis in Patients with Systemic Mastocytosis: A Monocentric Expert Centre Experience. Ann. Rheum. Dis..

[69] Weide R., Ehlenz K., Lorenz W., Walthers E., Klausmann M., Pflüger K.-H. (1996). Successful Treatment of Osteoporosis in Systemic Mastocytosis with Interferon Alpha-2b. Ann. Hematol..

[70] Lehmann T., Beyeler C., Lämmle B., Hunziker T., Vock P., Olah A.J., Dahinden C., Gerber N.J. (1996). Severe osteoporosis due to systemic mast cell disease: Successful treatment with interferon alpha-2B. Rheumatology.

[71] Kendler D.L., Cosman F., Stad R.K., Ferrari S. (2022). Denosumab in the Treatment of Osteoporosis: 10 Years Later: A Narrative Review. Adv. Ther..

[72] Hanley D.A., Adachi J.D., Bell A., Brown V. (2012). Denosumab: Mechanism of Action and Clinical Outcomes. Int. J. Clin. Pract..

[73] Bone H.G., Wagman R.B., Brandi M.L., Brown J.P., Chapurlat R., Cummings S.R., Czerwiński E., Fahrleitner-Pammer A., Kendler D.L., Lippuner K. (2017). 10 Years of Denosumab Treatment in Postmenopausal Women with Osteoporosis: Results from the Phase 3 Randomised FREEDOM Trial and Open-Label Extension. Lancet Diabetes Endocrinol..

[74] Tsourdi E., Langdahl B., Cohen-Solal M., Aubry-Rozier B., Eriksen E.F., Guañabens N., Obermayer-Pietsch B., Ralston S.H., Eastell R., Zillikens M.C. (2017). Discontinuation of Denosumab Therapy for Osteoporosis: A Systematic Review and Position Statement by ECTS. Bone.

[75] Rossini M., Zanotti R., Orsolini G., Tripi G., Viapiana O., Idolazzi L., Zamò A., Bonadonna P., Kunnathully V., Adami S. (2016). Prevalence, Pathogenesis, and Treatment Options for Mastocytosis-Related Osteoporosis. Osteoporos. Int..

[76] Orsolini G., Gavioli I., Tripi G., Viapiana O., Gatti D., Idolazzi L., Zanotti R., Rossini M. (2017). Denosumab for the Treatment of Mastocytosis-Related Osteoporosis: A Case Series. Calcif. Tissue Int..

[77] Sánchez López J.D., Cariati P., Pérez de Perceval Tara M. (2018). Osteonecrosis Maxilar Asociada a Tratamiento Con Denosumab En Una Paciente Con Mastocitosis Sistémica. Med. Clin..

[78] Joven M.H., Diemer K. (2025). MON-793 Long-Term Denosumab Treatment in a Patient with Low Bone Mass Associated with Indolent Systemic Mastocytosis. J. Endocr. Soc..

[79] Abbas M., Luthra P. (2025). Denosumab as a Potential Disease-Modifying Treatment in Indolent Systemic Mastocytosis-Related Osteoporosis. JCEM Case Rep..

[80] Gotlib J., Kluin-Nelemans H.C., George T.I., Akin C., Sotlar K., Hermine O., Awan F.T., Hexner E., Mauro M.J., Sternberg D.W. (2016). Efficacy and Safety of Midostaurin in Advanced Systemic Mastocytosis. N. Engl. J. Med..

[81] DeAngelo D.J., Radia D.H., George T.I., Robinson W.A., Quiery A.T., Drummond M.W., Bose P., Hexner E.O., Winton E.F., Horny H.-P. (2021). Safety and Efficacy of Avapritinib in Advanced Systemic Mastocytosis: The Phase 1 EXPLORER Trial. Nat. Med..

[82] George T.I., Karner K.H., Moser K.A., Rets A., Fredericks M., Reiter A., Radia D.H., Deininger M.W., Lin H.-M., Sen J. (2021). Efficacy of Avapritinib in Patients with Advanced Systemic Mastocytosis: Hematologic and Bone Marrow Responses from the Phase 2 Open-Label, Single-Arm, Pathfinder Study. Blood.

[83] Gotlib J., Reiter A., Radia D.H., Deininger M.W., George T.I., Panse J., Vannucchi A.M., Platzbecker U., Alvarez-Twose I., Mital A. (2021). Efficacy and Safety of Avapritinib in Advanced Systemic Mastocytosis: Interim Analysis of the Phase 2 PATHFINDER Trial. Nat. Med..

[84] Gotlib J., Castells M., Elberink H.O., Siebenhaar F., Hartmann K., Broesby-Olsen S., George T.I., Panse J., Alvarez-Twose I., Radia D.H. (2023). Avapritinib versus Placebo in Indolent Systemic Mastocytosis. NEJM Evid..

[85] Castells M., Sundaresh V., Gotlib J., Tashi T., Sabato V., Bulai Livideanu C., Akin C., Radia D., Siebenhaar F., Deangelo D. (2025). Changes in Long-Term Bone Health in Patients Receiving Avapritinib for the Treatment of Indolent Systemic Mastocytosis in the Pioneer Study. Blood.

[86] Reiter A., George T., Radia D., Deininger M., Luebke J., Lin H.-M., Bidollari I., Caetano-Lopes J., Yang G., King A. (2025). Avapritinib Treatment of Patients with Advanced Systemic Mastocytosis: 4-Year Safety, Effect on Bone and First-Line Efficacy Results of the Pathfinder Clinical Study. Blood.

[87] Nguyen A.H., Xiao W., Veletic I., Manshouri T., Braish J., El Hajjar G., Issa G., Pemmaraju N., Masarova L., Bose P. (2025). Clinical, Molecular, and Demographic Features of Systemic Mastocytosis: Analysis of a Large Cohort. Blood.

[88] Edwards A.M., Hagberg H. (2010). Oral and Inhaled Sodium Cromoglicate in the Management of Systemic Mastocytosis: A Case Report. J. Med. Case Rep..

[89] Soare D., Leru P., Bumbea H. (2025). Innovative Therapeutic Approaches in Systemic Mastocytosis: An Updated Review. Maedica—J. Clin. Med..

[90] Szudy-Szczyrek A., Bachanek-Mitura O., Gromek T., Chromik K., Mital A., Szczyrek M., Krupski W., Szumiło J., Kanduła Z., Helbig G. (2021). Real-World Efficacy of Midostaurin in Aggressive Systemic Mastocytosis. J. Clin. Med..

[91] Rossini M., Viapiana O., Zanotti R., Tripi G., Perbellini O., Idolazzi L., Bonifacio M., Adami S., Gatti D. (2015). Dickkopf-1 and Sclerostin Serum Levels in Patients with Systemic Mastocytosis. Calcif. Tissue Int..

[92] Robinson M.K., Caminis J., Brunkow M.E. (2013). Sclerostin: How Human Mutations Have Helped Reveal a New Target for the Treatment of Osteoporosis. Drug Discov. Today.

[93] Reid I.R. (2017). Targeting Sclerostin in Postmenopausal Osteoporosis: Focus on Romosozumab and Blosozumab. BioDrugs.

[94] Cosman F., Crittenden D.B., Adachi J.D., Binkley N., Czerwinski E., Ferrari S., Hofbauer L.C., Lau E., Lewiecki E.M., Miyauchi A. (2016). Romosozumab Treatment in Postmenopausal Women with Osteoporosis. N. Engl. J. Med..

[95] Chavassieux P., Chapurlat R., Portero-Muzy N., Roux J.-P., Garcia P., Brown J.P., Libanati C., Boyce R.W., Wang A., Grauer A. (2019). Bone-Forming and Antiresorptive Effects of Romosozumab in Postmenopausal Women with Osteoporosis: Bone Histomorphometry and Microcomputed Tomography Analysis After 2 and 12 Months of Treatment. J. Bone Miner. Res..

[96] Luebke J., George T.I., DeAngelo D.J., Gotlib J., Radia D.H., Deininger M.W., Zakharyan A., Caetano-Lopes J., Dimitrijevic S., Reiter A. (2024). Disease-Modifying Effects of Avapritinib in Patients with Advanced Systemic Mastocytosis: Improvements in Bone Density. Blood.

[97] Zhang Y., Ma M., Huang X., Liu J., Tian C., Duan Z., Fu H., Huang L., Geng B. (2025). Machine Learning Is Changing Osteoporosis Detection: An Integrative Review. Osteoporos. Int..

[98] Gattani A., Dixit S., Patil M., Gupta M., Navghane A., Hule O., Srinivasan K. (2026). Artificial Intelligence for Fall Detection in Older Adults: A Comprehensive Survey of Machine Learning, Deep Learning Approaches, and Future Directions. Ageing Res. Rev..

[99] Qin H., Ding Y., Ju J., Qu Z., Peng L. (2026). Enhanced fracture detection on radiographs with AI assistance for clinicians: A systematic review and meta-analysis. Ann. Med..

